# Does MHC heterozygosity influence microbiota form and function?

**DOI:** 10.1371/journal.pone.0215946

**Published:** 2019-05-16

**Authors:** M. A. Wadud Khan, W. Zac Stephens, Ahmed Dawood Mohammed, June Louise Round, Jason Lee Kubinak

**Affiliations:** 1 Department of Biology, University of Texas at Arlington, Arlington, Texas, United States of America; 2 Department of Pathology, University of Utah, Salt Lake City, Utah, United States of America; 3 Department of Pathology, Microbiology, and Immunology, University of South Carolina School of Medicine, Columbia, South Carolina, United States of America; University of Illinois at Urbana-Champaign, UNITED STATES

## Abstract

MHC molecules are essential for the adaptive immune response, and they are the most polymorphic genetic loci in vertebrates. Extreme genetic variation at these loci is paradoxical given their central importance to host health. Classic models of MHC gene evolution center on antagonistic host-pathogen interactions to promote gene diversification and allelic diversity in host populations. However, all multicellular organisms are persistently colonized by their microbiota that perform essential metabolic functions for their host and protect from infection. Here, we provide data to support the hypothesis that MHC heterozygote advantage (a main force of selection thought to drive MHC gene evolution), may operate by enhancing fitness advantages conferred by the host’s microbiome. We utilized fecal 16S rRNA gene sequences and their predicted metagenome datasets collected from multiple MHC congenic homozygote and heterozygote mouse strains to describe the influence of MHC heterozygosity on microbiome form and function. We find that in contrast to homozygosity at MHC loci, MHC heterozygosity promotes functional diversification of the microbiome, enhances microbial network connectivity, and results in enrichment for a variety of microbial functions that are positively associated with host fitness. We demonstrate that taxonomic and functional diversity of the microbiome is positively correlated in MHC heterozygote but not homozygote animals, suggesting that heterozygote microbiomes are more functionally adaptive under similar environmental conditions than homozygote microbiomes. Our data complement previous observations on the role of MHC polymorphism in sculpting microbiota composition, but also provide functional insights into how MHC heterozygosity may enhance host health by modulating microbiome form and function. We also provide evidence to support that MHC heterozygosity limits functional redundancy among commensal microbes and may enhance the metabolic versatility of their microbiome. Results from our analyses yield multiple testable predictions regarding the role of MHC heterozygosity on the microbiome that will help guide future research in the area of MHC-microbiome interactions.

## Introduction

MHC molecules display protein antigens (peptides) on the surface of cells and are central to the development of the vertebrate adaptive immune response. They are also important signaling molecules that coordinate immune cell development and effector function[1–6]. MHC genes are the most polymorphic loci known in vertebrates, and much of this polymorphism lies in the exons encoding the peptide-binding cleft of MHC molecules or the promoter regions of MHC genes[7–10]. MHC class I molecules are expressed on all nucleated cells and present intracellularly-derived peptide antigens to circulating cytotoxic T cells. These molecules are central to anti-viral and anti-tumor immunity. MHC class II molecules (MHCII) have more restricted expression and present extracellularly-derived peptides to circulating helper T cells. These molecules are central to humoral (i.e. antibody-mediated) immunity against extracellular microbes (primarily bacteria).

Several hypotheses have been put forth to explain the force of selection driving the evolution and maintenance of high allelic diversity at MHC loci and these can broadly be separated into two non-mutually exclusive mechanisms of diversifying selection; diversifying selection driven by antagonistic host-pathogen interactions and diversifying selection driven by inbreeding avoidance[8, 11, 12]. Heterozygote advantage (overdominance) is a pathogen-centric model of MHC evolution that is based on the argument that MHC heterozygotes have a more effective immune response than MHC homozygotes and consequently heightened resistance to infection. This phenomenon has been supported using epidemiological data from humans infected with viruses including Hepatitis B[13], HIV[14, 15], and HTLV[16]. Experimental infection studies in mice[17, 18] and studies across a diverse array of wild vertebrate species[19–23] also support the argument that MHC heterozygosity can enhance resistance to infectious disease. Because MHC genes are co-dominantly expressed enhanced resistance can arise because the immune system can mount responses against a more diverse array of pathogen epitopes, and because deleterious MHC alleles can be masked in the heterozygous condition[11, 18].

Hypotheses regarding the process of MHC gene evolution have focused on the fitness consequences associated with infection by pathogens. However, multicellular organisms are also stably colonized throughout their lives by a vast array of microbial species (collectively termed the ‘microbiota’) that provide essential metabolic services to their host[24] and enhance resistance to infection[25]. Moreover, previous empirical studies in animal models have demonstrated an association between MHC polymorphism and microbiota composition. Initial studies used liquid gas chromatography to compare short-chain fatty acid (SCFA) profiles (SCFAs are exclusively produced by bacterial symbionts in the gut) demonstrated differences in the composition of fecal flora among MHC congenic mouse strains[26, 27]. A subsequent study using bacterial 16S rRNA gene sequencing in stickleback fish, which are a classic model for studying MHC polymorphism, demonstrated associations between MHC alleles and microbial composition in the gut[28]. Importantly, this study was also the first to report an effect of MHC heterozygosity on the relative abundance of specific microbial species in the gut. A more recent study by Kubinak et al. (2015) used 16S rRNA sequencing to demonstrate that MHCII^-/-^ mice had a distinct microbial community form in their gut compared to WT C57BL/6 control animals, that microbial communities were taxonomically distinct among MHC congenic mouse strains reared under a highly controlled environment, and that the effect of MHC on microbiota composition was most pronounced on mucosally-associated gut microbial communities[1].

MHC molecules regulate vertebrate adaptive immunity and are encoded by the most polymorphic genes known. Is MHC polymorphism favored because it helps establish and maintain a physiologically beneficial microbiome? Here, we provide a dataset that addresses the hypothesis that MHC heterozygosity influences taxonomic composition and functional gene content of the microbiome. Here, we aimed at exploring the link between the MHC-modulated microbiota and host fitness by comparing predicted metabolic pathways. We show that MHC heterozygotes develop microbiomes that are enriched in microbial functions positively associated with host health. Results described here suggest that MHC heterozygosity may confer a fitness advantage by promoting the development of gut microbiome that is less invasive/pro-inflammatory, more metabolically capable, more resistant to invasion by pathogens, and/or more functionally adaptive.

## Materials and methods

### Ethics statement

All mice were reared at the University of Utah School of Medicine, and all mouse work adhered strictly to University and Federal guidelines regarding the use of animals in research. Animals were euthanized via CO2 asphyxiation. All animal work was approved by the University of Utah Institutional Animal Care and Use Committee (Protocol#14–05009).

### Animals

In this study, we utilized thirty 8–12 week old female BALB/c MHC congenic mice representative of three MHC homozygote (H2^*b/b*^, H2^*d/d*^, H2^*k/k*^) and three MHC heterozygote (H2^*b/d*^, H2^*d/k*^, H2^*k/b*^) genotypes (n = 5 female animals per genotype). We chose to limit our analysis to females due to the effect of sex on microbiota composition[29]. Multiple generations of each genotype used in this study were reared and maintained in the same facility under standardized environmentally-controlled conditions (IVC caging, automatic water, fed a standardized soy-free irradiated mouse chow (Envigo 2920X), 12:12 light:dark cycles). More specifically, in an effort to minimize the confounding effect of strain isolation, MHC homozygote animal purchased from Jackson Laboratories were used as founders to produce MHC heterozygote F_1_ progeny. F_1_ progeny were used to re-derive MHC homozygote F_2_ progeny. F_2_ MHC homozygote progeny were then used to produce the F_3_ progeny MHC homozygote and heterozygote animals used for this study (S1 Fig). To equalize cage effect, five female mice from each genotype were derived from a single breeding cage and came from the same litter. Thus, fifteen homozygote mice were derived from three separate cages and fifteen heterozygote mice were derived from three separate cages. Therefore, cage effect should not be a primary driver of observed differences between MHC homozygote and heterozygote mice. All mice were reared at the University of Utah School of Medicine, and all mouse work adhered strictly to University and Federal guidelines regarding the use of animals in research.

### 16S rRNA sequencing

Fecal samples were collected, immediately flash-frozen in liquid nitrogen, and stored at -80°C until downstream processing. DNA was extracted from fecal pellets using a bead-beating method following kit instructions (MoBio PowerFecal DNA Extraction Kit). Primers targeting the V3/V4 region of the bacterial 16S rRNA gene were used to generate PCR products for use in library prep. A detailed description of library preparation (including primer description) can be found in Kubinak et al.[1]. After quality filtering, all samples were rarified to a depth of 16,000 high quality sequence reads per sample for use in all of the analyses contained in this manuscript.

### Sequence processing and analysis

QIIME 1.8.0 software was used for the upstream and downstream processing of the raw 16S rRNA gene sequences^36^. Followed by primer truncating and quality filtering, the sequences were assigned to operational taxonomic units (OTUs) at a minimum of 97% sequence similarity using closed reference OTU-picking method. The upstream processing steps include the removal of sequences with anonymous bases, chimera sequences using vsearch method^82^. In addition, phred quality score of at least 30 was used to maintain high quality sequences. The GreenGenes database^41^ was used to cluster the quality-filtered reads using Uclust algorithm^40^, and then RDP classifier^42^ was used to assign taxonomy. Before analysis, we performed following filtering strategies on the OTU-table: (1) singleton OTUs were removed to reduce the possibility of sequencing errors, (2) all samples were rarified to 16,000 sequences to avoid sequence effort bias. PICRUSt (Phylogenetic Investigation of Communities by Reconstruction of Unobserved States) analysis, which is a predictive metagenomics analysis that utilizes 16S rRNA sequencing data, was used to infer functional gene content^37^. A Bray-Curtis (BC) metric was used for all beta-diversity analyses comparing community diversity between MHC homozygotes and MHC heterozygotes. Phylogenetic (UniFrac) comparisons among genotypes are provided as supplemental data (S2 Fig). We use the terms "overall" and "core" to refer to all observed OTUs among mice or the subset of OTUs shared among all (100%) of samples, respectively. Alpha diversities were calculated to compare the amount of diversity contained within MHC heterozygotes and MHC homozygotes using Shannon index, a taxonomic metric and phylogenetic distance, a phylogenetic metric. A richness metric was used to quantify the total number of observed functional genes.

### Network construction

Networks for each genotype were constructed to identify correlations among members of bacterial communities, where relative OTU abundance data was used to calculate Spearman correlation coefficients between all possible pairs of OTUs across mice. OTU-pairwise correlations between these OTUs were performed on within-group data. OTU-pairs with correlation coefficient values of either >0.8 or <-0.8, and corrected *P* ≤ 0.05 (Benjamini-Hochberg procedure) were used in the construction of networks. All OTU-pairs that did not satisfy these criteria were discarded from the analysis. The analysis was performed in R environment using “*multtest*” package^38^, and networks were plotted using Cytoscape v3.2.1^39^. Interacting OTUs within networks represent significant correlations across samples, where the positive correlations indicate the similar abundance pattern of the interacting OTUs, while negative correlations signify opposite abundance pattern. Following construction, we characterized and compared networks by calculating few of their topological features: (1) number of nodes, which represents the total number of OTUs in the network; (2) number of edges, which represents the total number of significant pairwise correlations within a network; (3) connectivity, which is a ratio between number of edges and number of nodes in a network; (4) betweenness centrality, which represents the importance of a node by estimating the number of times it acts as a connector along the shortest path between two other nodes within a network. Therefore, higher betweenness centrality value of a node is indicative of its higher involvement in the overall connectivity with other nodes in the network, and vice versa.

### Statistics

We employed DESeq2 algorithm^80^, an R package, to identify the fecal microbial taxa and PICRUSt-predicted functional KEGG Orthology (KO) genes that are differentially abundant between homozygotes and heterozygotes. Compared to the abundances of the microbial taxa and predicted KO genes in the fecal microbial community of homozygotes, this test identified whether they are differentially abundant in the heterozygote communities. The differences of their abundances were reported as log_2_FoldChange, where features (i.e., taxa and KO genes) enriched in homozygote are indicated by positive values, and heterozygote enriched features have negative values. To compare the alpha diversities, and pairwise taxonomic distances between samples, a nonparametric two-sided two sample *t*-test was carried out using 999 Monte Carlo permutations. In these statistical tests, Benjamini-Hochberg procedure was used to adjust the *P*-values, and statistical significance was considered at adjusted *P* < 0.05. Error bars represent standard errors of mean (S.E.M). Mantel test was performed to test the correlation of Bray-Curtis distances between taxonomic and functional gene contents within homozygote and heterozygote communities, where 999 permutations were used to calculate the *P*-values. Analysis of similarity (ANOSIM) test was used for multivariate comparisons shown in all PCoA plots. Area under curve (AUC) analysis was performed to compare the maintenance of taxonomic and functional gene content across MHC homozygote and MHC heterozygote animals. The areas under the curves were calculated in GraphPad Prism version 7, and a two-tail *P*-value was estimated as described elsewhere^81^. Unless otherwise stated, all statistical analyses were performed in QIIME 1.8.0 and 1.9.0 versions.

## Results

### Diversity patterns of fecal bacterial community

**Fig 1:**
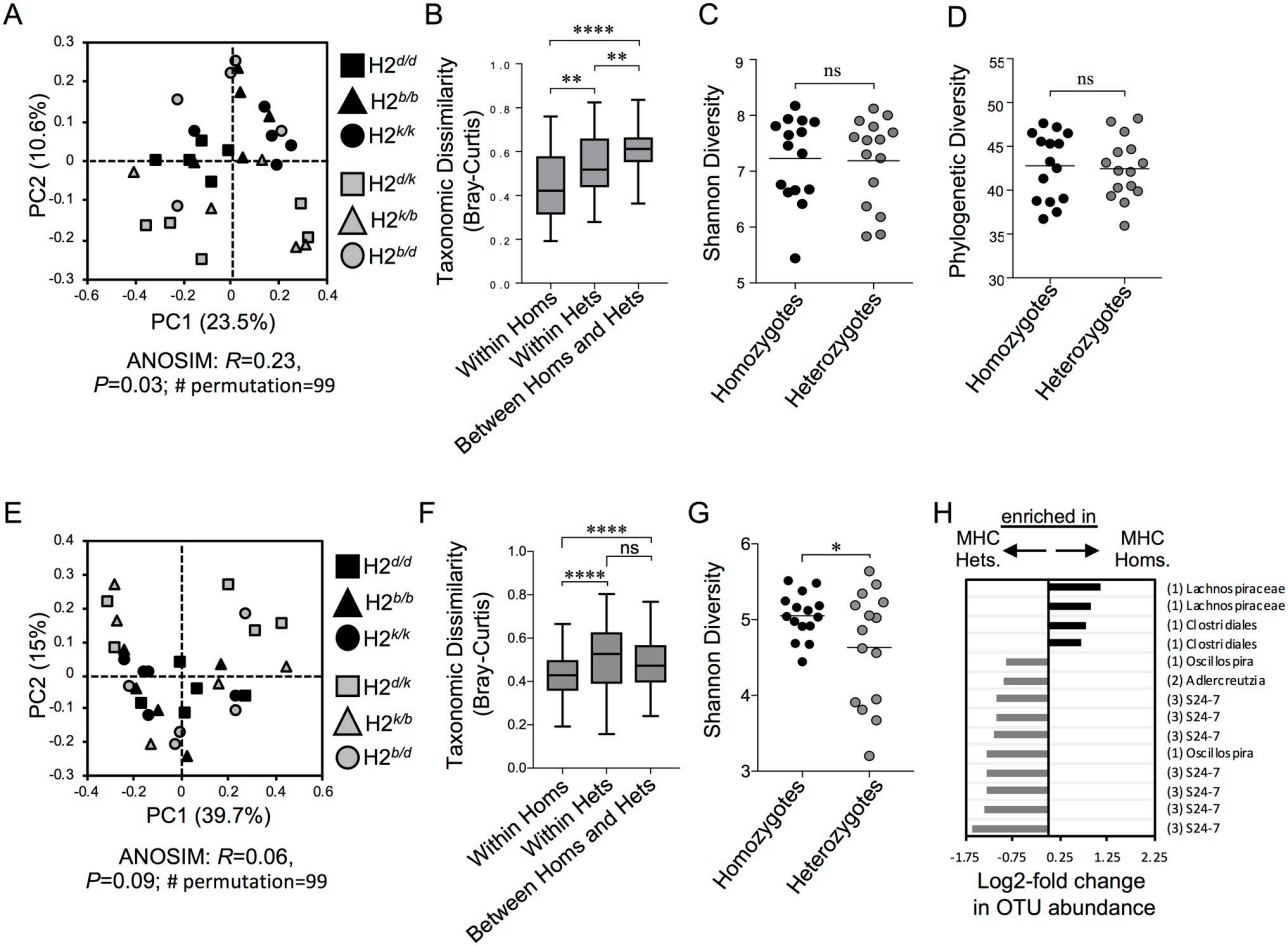
MHC heterozygosity alters microbiota composition and drives community divergence among individuals. **A)** PCoA of beta-diversity comparison of overall community taxonomic composition between MHC homozygote and MHC heterozygote animals. Bray-Curtis analysis, ANOSIM: *R* = 0.23; *P* = 0.03; no. of permutations = 99. **B)** Boxplots representing within-group taxonomic dissimilarity and taxonomic dissimilarity comparisons between MHC homozygote and MHC heterozygote overall community. Comparison of alpha diversities in overall communities between MHC homozygote and MHC heterozygote animals based on Shannon **(C)**, and phylogenetic diversity **(D)** indices. **E)** PCoA of beta-diversity comparison of core community between MHC homozygote and MHC heterozygote animals. Bray-Curtis analysis, ANOSIM: *R* = 0.06; *P* = 0.09; no. of permutations = 99. **F)** Boxplots representing within-group taxonomic dissimilarity and taxonomic dissimilarity comparisons between MHC homozygote and MHC heterozygote core community. **G)** Comparison of alpha diversities in core communities between MHC homozygote and MHC heterozygote animals based on Shannon diversity index. **H)** Differential enrichment in abundance of specific taxa within the core microbiota of MHC homozygote and MHC heterozygote animals. The significance threshold in this plot was *P* <0.05, Benjamini-Hochberg corrected. X-axis shows the values of log_2_FoldChange, where positive values indicate the taxa enriched in homozygotes (black) and negative values indicate the taxa enriched in heterozygotes (grey). 1 = *Firmicutes* (*Clostridiales*); 2 = *Actinobacteria*; and 3 = *Bacteroidetes*. Symbols (*), (**), (***), and (****) indicate the Benjamini-Hochberg corrected significance values of *P* < 0.05, *P* < 0.01, *P* < 0.001, *P* < 0.0001 respectively, which were calculated using non-parametric two sample *t*-test.

16S rRNA microbial community profiling was performed to test whether MHC genotype impacts taxonomic composition of the microbiota. "Overall" (i.e. all observed OTUs) microbiota community diversity was significantly different between MHC homozygotes and heterozygotes (Fig 1A; ANOSIM: *R* = 0.23, *P* = 0.03). Overall microbiota composition was significantly more dissimilar among MHC heterozygous animals compared to homozygotes (Fig 1B; non-parametric two-sample *t*-test: Benjamini-Hochberg adjusted *P* < 0.01). There was no observed difference in alpha diversity measures (Shannon diversity (Fig 1C) and phylogenetic diversity (Fig 1D); adjusted *P* > 0.05 in both cases) between overall homozygote and heterozygote communities. Within the overall communities of 692 OTUs, we tested for differences in OTU abundance between samples in the homozygotes and heterozygotes, and estimated that 98 OTUs are differentially abundant in homozygous animals, whereas 91 OTUs are differentially abundant in heterozygous animals (S1 Table; DESeq2 test: Benjamini-Hochberg adjusted *P* < 0.05). Most of the differentially abundant taxa belong to the *Firmicutes* phylum.

The concept of a “core microbiota” is based on the idea that there is a subset of microbial species (or functions) within complex communities that play fundamental roles in ecosystem function. Their fundamental role in ecosystem function would predict that these species should disproportionately influence host fitness. Therefore, we wanted to focus a portion of our analyses on the influence of MHC heterozygosity on the functional capacity of the core microbiota. We strictly defined core microbiota membership as those species present in all of the mice used in this study. Of the 692 total OTUs identified in our analysis, approximately 12% (82 OTUs) were found in all mice. Predictably, there is no gross difference in taxonomic composition of the core microbiome between MHC heterozygote and MHC homozygote animals (Fig 1E; ANOSIM: *R* = 0.06, *P* = 0.09). However, we noticed, in comparison to the heterozygotes, that the core microbial community of homozygotes showed significantly lower pairwise taxonomic dissimilarity, indicating any two homozygote communities are more similar in taxonomic composition (Fig 1F; adjusted *P* < 0.0001). MHC homozygotes also had higher Shannon diversity, indicating that the relative abundance of core species was significantly less variable (uneven) among MHC homozygotes compared to heterozygotes (Fig 1G; adjusted *P* < 0.05). Within the core microbiota of MHC heterozygotes, specific species within the S24-7 family (phylum *Bacteroidetes*), Oscillospira genus, and Adlercreutzia genus are enriched in abundance (Fig 1H; DESeq2 test: Benjamini-Hochberg adjusted *P* < 0.05). Within the core microbiota of MHC homozygotes, specific species within the Lachnospriaceae family and Clostridiales Order were enriched in abundance (Fig 1H; DESeq2 test: Benjamini-Hochberg adjusted *P* < 0.05).

### Diversity patterns of predicted functional gene profiles

**Fig 2:**
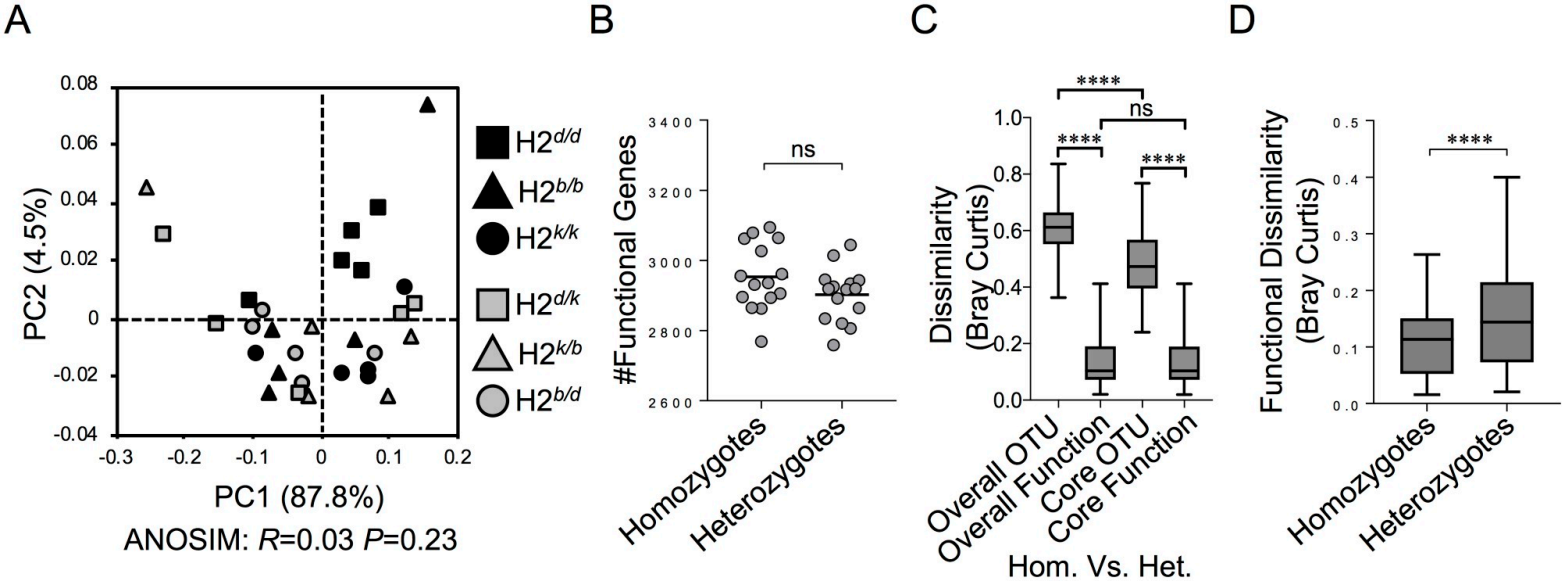
MHC heterozygosity drives functional divergence among individuals. **A)** PCoA of functional beta-diversity comparison between MHC homozygote and MHC heterozygote animals based on Bray-Curtis analysis of PICRUSt-annotated KEGG Orthology (KO) genes (ANOSIM: *R* = 0.03; *P* = 0.23; no. of permutations = 99). **B)** The number of functional genes is compared between MHC homozygote and MHC heterozygote animals. **C)** Boxplots representing within-group functional dissimilarity comparisons between MHC homozygotes and MHC heterozygotes based on Bray-Curtis analysis. **D)** Boxplots representing within-group predicted KO gene dissimilarity between MHC homozygote and MHC heterozygote animals based on Bray-Curtis analysis. Symbol (****) indicates Benjamini-Hochberg corrected significance values of *P* < 0.0001, which were calculated using non-parametric two sample *t*-test.

We next performed PICRUSt analysis using 16S rRNA sequencing data as a method to infer whether functional gene diversity was influenced by MHC heterozygosity. In contrast to taxonomic diversity, the composition of functional genes contained within overall microbiota communities was not a discriminating factor between homozygotes and heterozygotes (Fig 2A; ANOSIM: *R* = 0.03, *P* = 0.23). Similarly, the total number of inferred functional genes was consistent between genotypes when considering the overall microbiome (Fig 2B; adjusted *P* > 0.05). The total number of observed functional genes were equivalent between overall and core microbiomes despite the core microbiota representing only 12% of the total observed species diversity observed (not shown). This result is due to bias in PICRUSt analysis that arises because quantification of inferred functions is based on an extremely limited number of known microbial genes. It is important to acknowledge this sampling bias here, because we do not want the reader to erroneously conclude that non-core species do not contribute novel functional genes. When comparing the degree of dissimilarity between MHC homozygote and MHC heterozygote microbial communities based on taxonomic and functional composition, we found that taxonomic dissimilarity between homozygotes and heterozygotes was significantly reduced when considering the core versus overall microbiota (Fig 2C; adjusted *P* < 0.0001). This makes sense based on how we have defined our core microbiota; the OTUs that are observed in all samples. Because the same species make up the core MHC homozygote and MHC heterozygote communities, this will decrease taxonomic dissimilarity between these communities. However, overall and core functional dissimilarity was the same between MHC homozygotes and heterozygotes despite exclusion of approximately 90% of the total observed species from the core microbiota. Again, this is explained by the sampling bias mentioned above and should not be interpreted as biologically meaningful. Finally, variability in gene content of the overall microbiome was significantly higher among MHC heterozygotes compared to homozygotes (Fig 2D; adjusted *P* < 0.0001).

### MHC heterozygosity is associated with less functional redundancy in the microbiome

**Fig 3:**
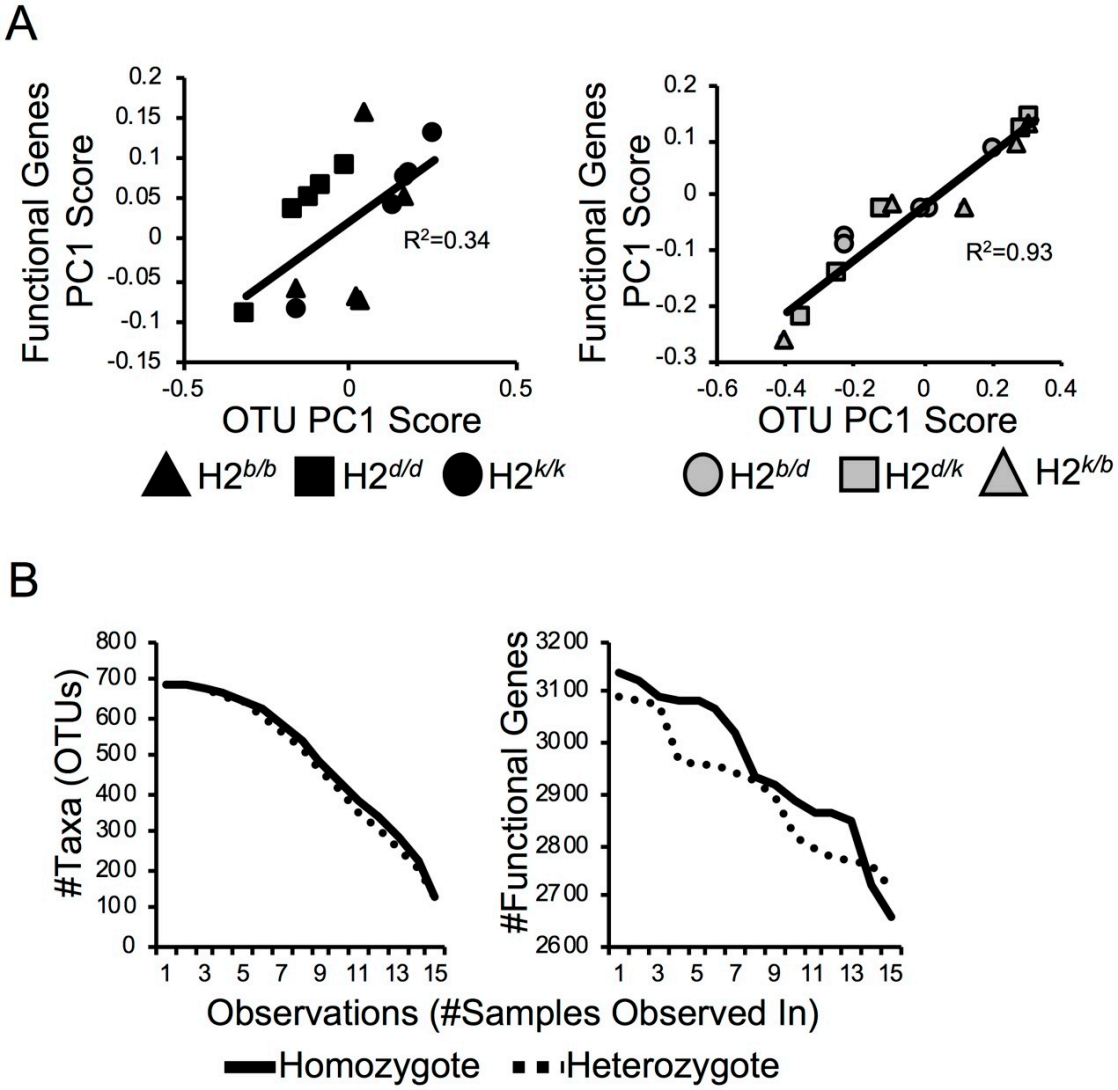
MHC heterozygotes have a less functionally redundant microbiome. **A)** Relationships of beta-diversity (Bray-Curtis) patterns between closed-referenced OTUs and predicted functional (KO) genes within MHC homozygotes and MHC heterozygotes. In this analysis, the beta diversity patterns are shown using the principal coordinates scores of the first axes of taxonomic and predicted KO gene datasets. Mantel tests conducted to estimate the significance of the correlation (Homozygotes: *r* = 0.39, *P <* 0.01; Heterozygotes: *r* = 0.71, *P <* 0.01). **B)** Decay plots illustrate the loss of taxonomic and functional diversity across MHC homozygote and MHC heterozygote animals. *P*-value in based on area under curve (AUC) analysis. Area under the curve of homozygote and heterozygote taxonomic datasets are estimated to be 6,759 and 6,974, respectively, where this difference is not statistically significant. On the other hand, AUCs of homozygote and heterozygote PICRUSt-annotated datasets are estimated to be 41,407 and 40,632, respectively, where this difference turns out to be significantly different (*P* < 0.01).

We next quantified the relationship between taxonomic and functional diversity in the overall MHC homozygote and MHC heterozygote microbiomes. Using correlation analysis, we found a stronger linear relationship between taxonomic and functional diversity in MHC heterozygotes compared to MHC homozygotes (Fig 3A; Mantel test: *r* = 0.39, *P <* 0.01 for homozygotes; *r* = 0.71, *P <* 0.01 for heterozygotes). Moreover, when we considered the loss of functional diversity across individuals (i.e. functional decay) we also found that gene functions were more rapidly lost among MHC heterozygote than MHC homozygote animals (Fig 3B, right panel), while taxonomic diversity was lost at a similar rate between groups (Fig 3B, left panel). Collectively, these data suggest that the observed reduction in functional dissimilarity among MHC homozygote animals (Fig 2D) may be due to the fact that there is more functional redundancy among bacteria found in MHC homozygote compared to MHC heterozygote microbiomes.

### Genotype-level network analyses predict more complex interactions within heterozygote core bacterial communities

In an ecosystem, microorganisms do not remain isolated, they interact with each other and form a complex web of metabolic interactions. The functional capacity of a microbial community is influenced by the degree to which metabolically active species interact to form trophic cascades^43,44^. It is therefore assumed that microbial species that feed off of products produced by other species (syntrophic interactions), have positively correlated abundance patterns. Alternatively, when two microbes interact in an antagonistic manner (e.g. through the production of antimicrobials), they will have inversely correlated abundance patterns. Network analysis is a method used to characterize the general phenotype of species interactions in an ecosystem. We wanted to determine whether MHC heterozygosity could influence the structure of microbial networks. For this analysis, we again focused on the core microbiota for two reasons. First, these species are hypothesized to contribute most to the essential metabolic functions of the host. Second, peripheral species are transient (or rare) microbes that either may not form stable interactions with other species or may not provide essential functions to the host. Third, we wanted to reduce genotype-specific effects (i.e. the presence of bacterial species, and their associated functions, that are only found in certain homozygote or heterozygote genotypes) in order to focus on differences in more generalizable features of the microbiome across our six genotypes.

**Fig 4:**
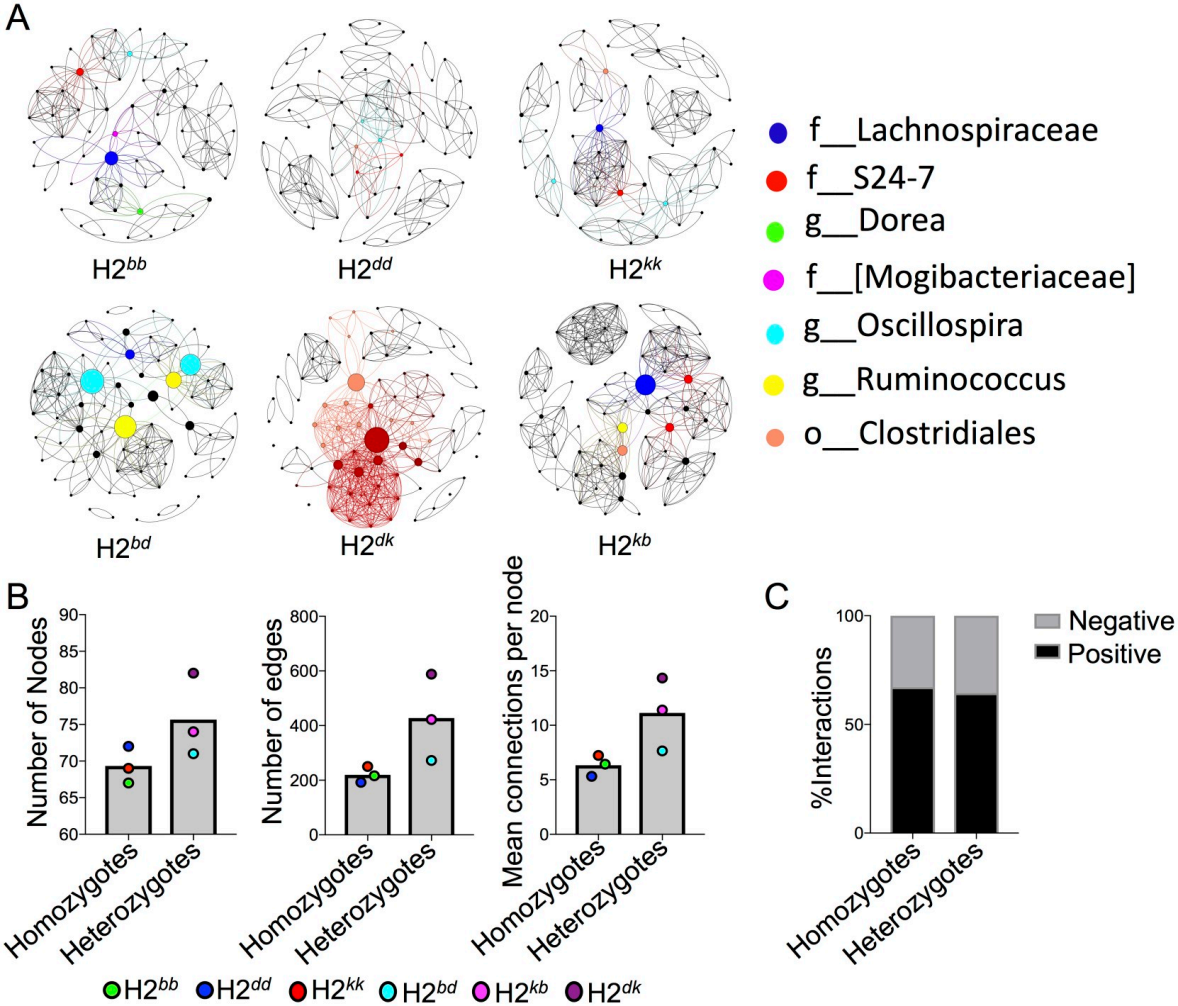
MHC heterozygotes have a more connected microbial network in core communities. **A)** Plots depict association between microbial species using Spearman correlation coefficient values of >0.8 for positive correlations, and <-0.8 for negative correlations with statistical significance *P*<0.01, Benjamini-Hochberg corrected. The microbial pairs that follow these criteria were considered to have strong association among them and included in network study. In the network analysis, nodes represent microbial taxa (OTUs), and lines (or edges) connecting the nodes represent significant pairwise correlations in species abundances. The size of the nodes is proportional to their betweenness centrality values. Nodes with highest betweenness centrality values (“hub” species; here we considered top seven OTUs across networks), along with their edges, are colored for comparative understanding of their connectedness across genotype-wise networks. These nodes are identified taxonomically in the accompanying legend. **B)** The comparative analysis of few network topologies between MHC homozygote and MHC heterozygote networks, number of nodes, number of edges, mean connection per node (i.e., ratio between number of edges and number of nodes in a network), and relative proportion of positive and negative correlations are illustrated.

We constructed genotype-level networks (Fig 4A), and compared between them by their topological features (Fig 4A and 4B). The total number of nodes (i.e. number of species having one or more interactions), significant OTU-OTU pairs (r > 0.8 or r < -0.8, Benjamini-Hochberg adjusted *P* ≤ 0.05) (edges), and the average number of edges per node (connectivity) were all higher in MHC heterozygotes compared to homozygotes (Fig 4B). Later, we investigated node-wise feature in the networks by their betweenness centrality values, and identified few nodes (i.e., OTUs) in each network that, by definition, are most important in connecting other OTUs in their respective networks (Fig 4A). Each genotype has a set of these nodes, which, in general, have higher betweenness centrality values in heterozygotes compared to homozygotes (S2 Table), suggesting these nodes are more important in heterozygotes as they more frequently connect other nodes in heterozygote networks and contribute to forming more complex networks compared to homozygotes. For example, an unclassified member of *Lachnospiraceae* (OTU ID # 258202) is the most important node in both H2^*kk*^ and H2^*kb*^ networks, where the betweenness centrality values are 0.071, and 0.024, respectively. We also analyzed the relative proportion of positive and negative correlations in the networks. Positive interactions dominated in both MHC heterozygote as well as MHC homozygote core microbiota (~70% positive correlations versus ~30% negative), and there was no difference in this ratio between groups (Fig 4C). Taken together, higher connectivity within heterozygote networks might represent a more complex web of metabolic interactions within core bacterial community, where nodes with high centrality values are assumed to play major metabolic hubs. These data support higher diversity of functional (KO) genes in relation to taxonomic (OTU) diversity (Fig 3), suggesting MHC heterozygosity favors metabolic versatility of the microbiome.

### Metabolic pathway analysis supports that MHC heterozygosity favors host-microbe symbiosis

Again, to focus on differences in microbiome functions generalizable across our six genotypes, we compared PICRUSt-predicted KEGG Orthology (KO) gene profiles of core microbial communities between homozygotes and heterozygotes and explored the alteration of specific functional genes and pathways caused by MHC heterozygosity. To check the accuracy of PICRUSt prediction, we calculated weighted Nearest Sequenced Taxon Index (weighted NSTI) scores for each sample that reflects the availability of sequenced genomes that are closely related to the OTUs in a given sample (S3 Table). A score of 0.2 indicates that functionality of microbial community in a given sample, on an average, can be predicted from reference genomes of similar species (80%). Since the close relatives of most of the core OTUs were not identified to the species level using the database used in our study (S2 Table), we obtained high NSTI scores, ranging 0.17 to 0.31.

**Fig 5:**
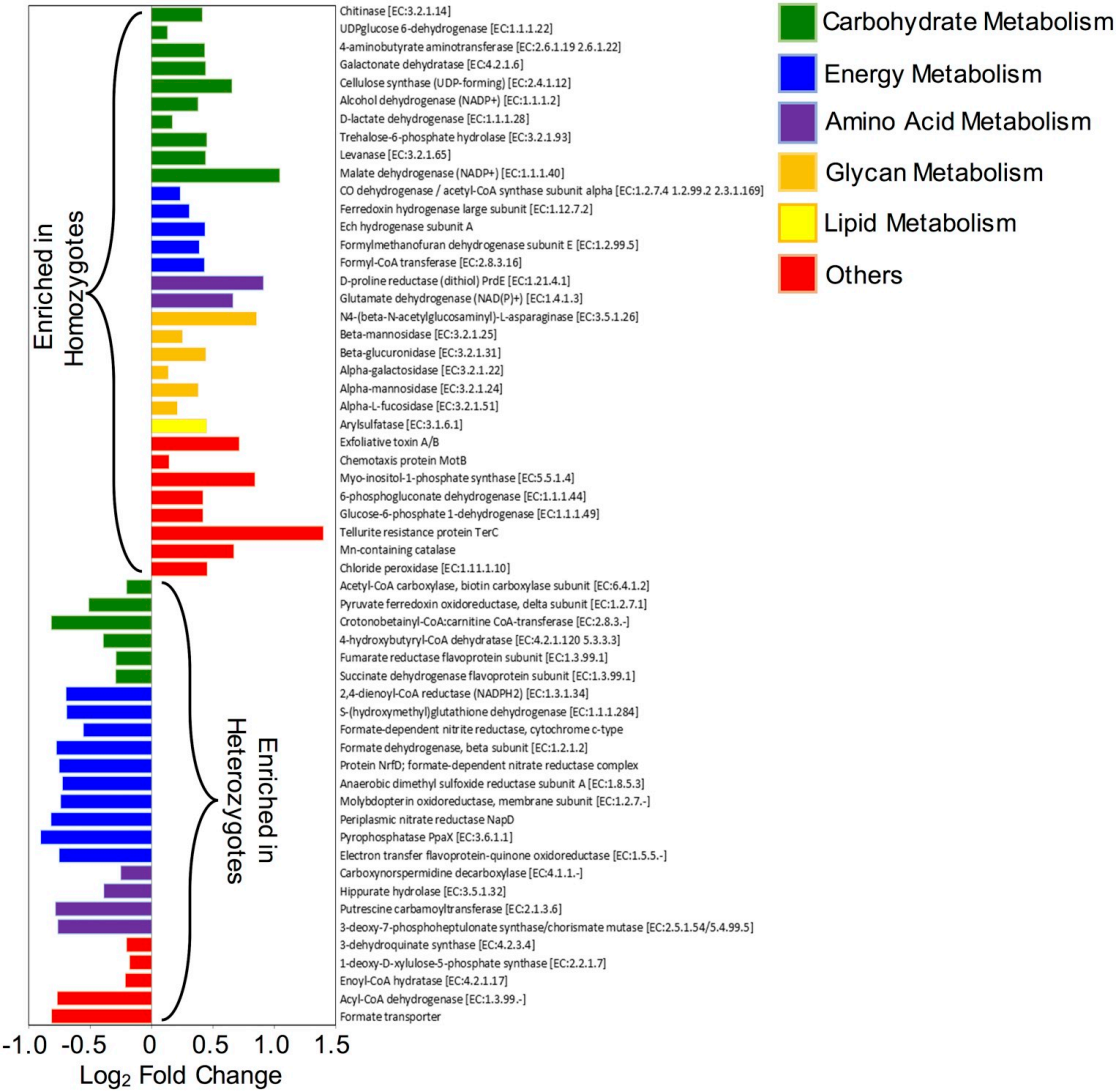
MHC heterozygosity influences core microbiome function. Major differentially abundant predicted KO genes encoding ECs involved in the KEGG functional categories between MHC homozygote and MHC heterozygote animals (see S4 Table for full list).X-axis shows the values of log_2_FoldChange, where positive values indicate the predicted KO genes enriched in MHC homozygotes, and negative values indicate the genes enriched in MHC heterozygotes. Bars are color coded by KEGG Level 2 functional groupings that are defined in the accompanying legend.

Numerous differences were found in the relative abundance of inferred functional genes between MHC heterozygote and homozygote core microbiomes (Fig 5; S4 Table; DESeq2 test: Benjamini-Hochberg adjusted *P* < 0.05). In the core microbiome of MHC homozygotes, we observed increased abundance of genes associated with the metabolism of host-derived carbohydrates that comprise the mucus layer overlaying gut epithelium (alpha-L-fucosidase [EC:3.2.1.51], alpha-galactosidase [EC:3.2.1.22], alpha-mannosidase [EC:3.2.1.24], and beta-mannosidase [EC:3.2.1.25]). Additionally, MHC homozygotes have higher representation of predicted genes involved in the degradation of other glycans such as dietary fibers (N4-(beta-N-acetylglucosaminyl)-L-asparaginase [EC:3.5.1.26] and levanase [EC:3.2.1.65]). MHC homozygotes were also observed to be enriched for genes associated with D-lactate metabolism (D-lactate dehydrogenase [EC:1.1.1.28]), acetylaldehyde production (alcohol dehydrogenase (NADP^+^) [EC:1.1.1.2]), antimicrobial production such as streptomycin (Myo-inositol-1-phosphate synthase [EC:5.5.1.4]), bacterial toxin production (exfoliative toxin A/B), bacterial motility (Chemotaxis protein MotB), and oxalate synthesis (Formyl-CoA transferase [EC:2.8.3.16]).

MHC heterozygote core microbiomes were enriched for genes associated with biosynthesis of essential amino acids like phenylalanine and tryptophan (3-phosphoshikimate 1-carboxyvinyltransferase [EC:2.5.1.19], 3-dehydroquinate synthase [EC:4.2.3.4], 3-deoxy-7-phosphoheptulonate synthase/chorismate mutase [EC:2.5.1.54 5.4.99.5], cinnamoyl-CoA:phenyllactate CoA-transferase [EC:2.8.3.17]), genes associated with the production and utilization of short-chain fatty acids (enoyl-CoA hydratase [EC:4.2.1.17], 2,4-dienoyl-CoA reductase (NADPH2) [EC:1.3.1.34], acetyl-CoA carboxylase [EC:6.4.1.2]), genes associated with the cellular respiration and ATP synthesis (pyruvate ferredoxin oxidoreductase, beta subunit [EC:1.2.7.1], succinate dehydrogenase flavoprotein subunit [EC:1.3.99.1], fumarate reductase flavoprotein subunit [EC:1.3.99.1], malate dehydrogenase (oxaloacetate-decarboxylating)(NADP+) [EC:1.1.1.40], pyrophosphatase PpaX [EC:3.6.1.1], flavoprotein-quinone oxidoreductase [EC:1.5.5.-], Protein NrfD; formate-dependent nitrate reductase complex), and genes associated with polyamine production (carboxynorspermidine decarboxylase [EC:4.1.1.-], putrescine carbamoyltransferase [EC:2.1.3.6]). Heterozygotes also have more genes associated with terpenoid backbone biosynthesis (1-deoxy-D-xylulose-5-phosphate synthase [EC:2.2.1.7], geranylgeranyl diphosphate synthase, type II [EC:2.5.1.1 2.5.1.10 2.5.1.29]).

**Fig 6:**
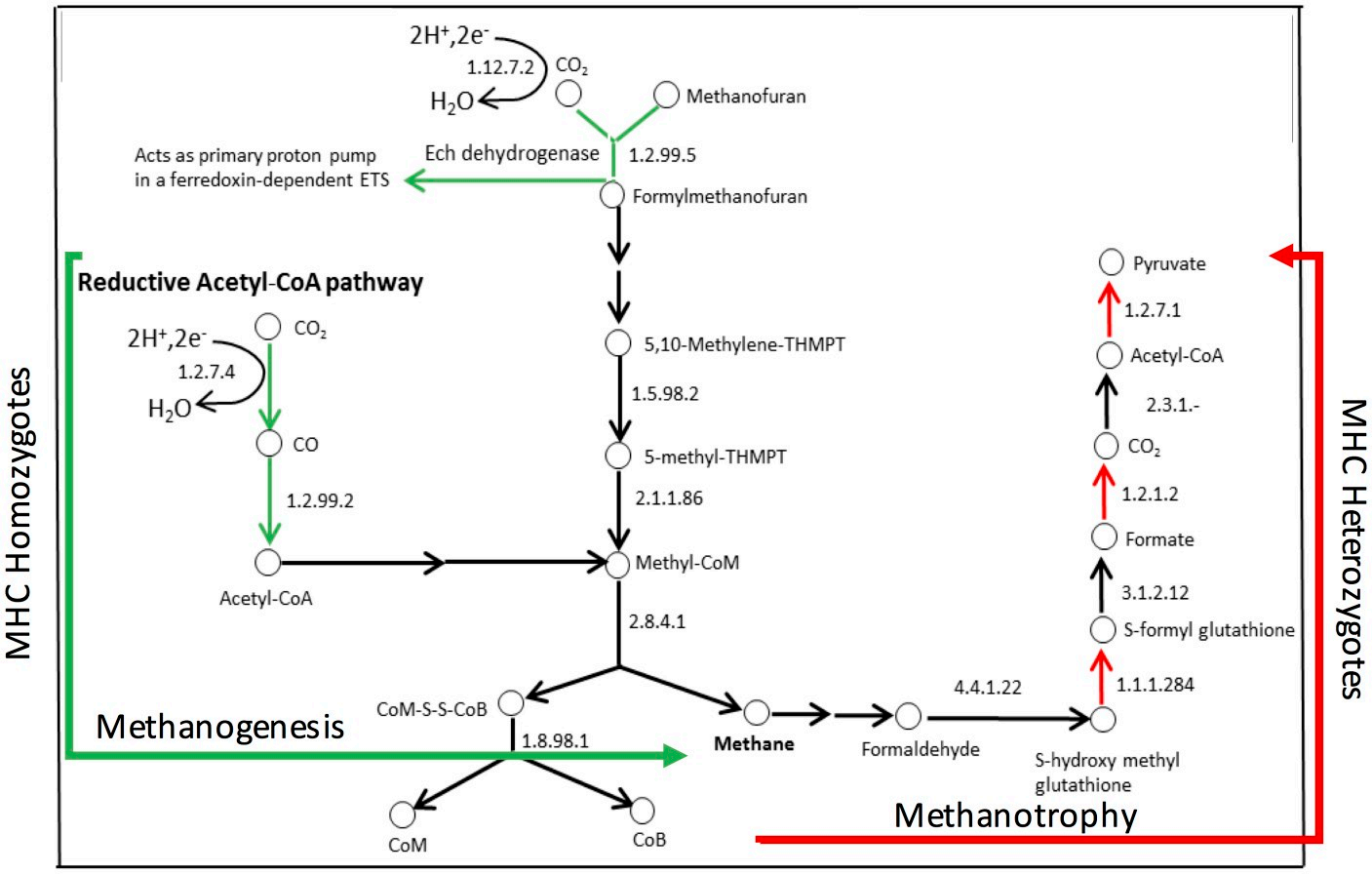
MHC heterozygosity impacts methane metabolism in the gut. Within the “core” microbial community of gut ecosystems, methanogenesis associated KO genes are enriched in MHC homozygotes, while MHC heterozygotes have higher abundance of KO genes associated with methanotrophic pathways. Green arrows indicate the genes enriched in MHC homozygote communities, while red arrows indicate the genes enriched in MHC heterozygote communities. Black arrows indicate either the observed genes are not differentially abundant between MHC homozygote or MHC heterozygote microbiomes (*P* > 0.05) or these genes are not included in PICRUSt database. The numbers in each step of the pathways indicates the EC number of associated enzymes.

Notably, we found one metabolic pathway that had opposing patterns of gene enrichment between MHC homozygotes and MHC heterozygotes, and it is associated with methane metabolism (Fig 6). MHC homozygotes were observed to be enriched for genes associated with methanogenesis (a byproduct of fermentation) (formylmethanofuran dehydrogenase subunit E [EC:1.2.99.5]), while MHC heterozygotes were enriched for genes associated with the utilization of methane as a carbon source (methanotrophy)(formate dehydrogenase, beta subunit [EC:1.2.1.2], formate dehydrogenase, gamma subunit [EC:1.2.1.2]).

## Discussion

Several major observations from our sequencing study support the hypothesis that MHC heterozygosity significantly influences microbiome form and function. First, MHC heterozygotes have a unique taxonomic composition of their gut microbiota compared to that of MHC homozygotes. Second, while the microbiomes of MHC heterozygotes have similar numbers of total gene functions compared to MHC homozygotes, heterozygosity results in significant differential enrichment for certain functions relevant to host health; notably within the core microbiome that is hypothesized to be particularly important to host fitness. In particular, the core microbiomes of MHC heterozygotes appear to be enriched for beneficial functions and deficient in functions that have been associated with a variety of gastrointestinal diseases. MHC heterozygosity also results in greater individual variation in taxonomic and functional composition of the microbiome, which could be linked to selection favoring the addition of species that perform novel functions. All else being equal, novel functions are more likely to come from more phylogenetically distinct species. Interestingly, we also found that the core microbiome of MHC heterozygotes tends to reflect a more integrated network of species.

Our results complement other studies that have linked MHC polymorphism with taxonomic differences in microbiota composition[26–28]. Most current efforts to understand the physiologic role of the microbiota have relied on taxonomic compositional descriptions like this, but the functions that these species perform, are what impacts host fitness directly. Therefore, we wanted to infer the functional relevance of observed taxonomic differences between MHC heterozygotes and homozygotes. While taxonomic diversity drove significant differences in overall microbiota composition (Fig 1A), functional diversity did not (Fig 2A). MHC heterozygotes and homozygotes contained similar numbers of functional genes (Fig 2B). This finding is consistent with another study demonstrating largely overlapping functionality of the microbiomes of diverse mammal species, despite significant taxonomic divergence[30]. However, despite possessing largely overlapping functional profiles, we observed differential enrichment of specific functional genes between MHC homozygotes and heterozygotes (discussed below). Overall, several observations support the hypothesis that MHC heterozygotes may be more functionally versatile and metabolically capable.

The relationship between taxonomic and functional diversity within microbiomes is influenced by the degree of functional redundancy that exists among microbial species[31]. This is an important relationship because it determines the degree to which species diversity impacts host fitness and can be acted upon by natural selection. Functional redundancy can be adaptive by increasing the stability of a microbiome (i.e. chance of losing a critical function is reduced), or maladaptive by decreasing functional versatility of the host (i.e. the capacity to acquire novel functions). One of the most interesting results from our analysis was the significant difference in the correlation between taxonomic and functional diversities between MHC homozygotes and heterozygotes (Fig 3A and 3B). Microbiomes among MHC heterozygotes (beta diversity) were more variable in taxonomic composition (Fig 1B) and functional gene content (Fig 2D) compared to MHC homozygotes. This is in contrast to the fact that MHC heterozygotes did not have on average more taxonomic (Fig 1C and 1D) or functional diversity (Fig 2B) within their communities (alpha diversity). We can infer two points from this observation. First, this means that increased inter-individual variation observed in MHC heterozygotes is not simply a consequence of heterozygotes being colonized by a wider variety of species. Second, it suggests that whatever mechanism drives the effect of MHC heterozygosity on microbiota composition, it is a diversifying force of selection that favors the addition of novel functions as microbial species are added to a community. In contrast, that same force of selection appears to drive communities towards a taxonomically and functionally more homogenous composition in MHC homozygotes. Throughout life, microbes with novel functions are ingested through the act of feeding. If these bacteria confer enhanced ability to extract nutrients from a particular food source, then we would hypothesize that the ability to integrate novel functions into an existing microbiome is a fitness enhancing trait. Future studies measuring fitness differentials between MHC homozygote versus MHC heterozygote animals fed simple to increasingly complex diets, or fed the same diet versus a diverse array of diets will be useful for addressing this.

Microorganisms in the gut form a complex network of metabolic interactions. Understanding the processes that shape these interactions is important because they are deterministic factors governing the adaptability and resilience of microbiomes in response to environmental variability. The shape of these interactions (i.e. network topology) may also be a diagnostic feature of certain disease states. Positive interactions between microbial species occur through nutritional cross feeding; the synthesis of molecules that enhance the fitness of other microbes. In contrast, negative interactions occur through the synthesis of antimicrobial molecules and environmental modifications (nutritional or immunological modifications) that inhibit the growth of other microbes. Here, we have provided for the first time an analysis of the potential impact of MHC polymorphism on gut microbiome network topology. Our data suggests that MHC heterozygotes have more potentially interacting species, more total interactions between species, and more interactions per species within their core microbiomes compared to MHC homozygotes (Fig 4). Network modularity (i.e. species clustering) is also more complex in MHC heterozygotes versus homozygotes, with those species possessing the highest betweenness centrality scores (i.e. metabolic “hub” species) represented by OTUs, closest relatives of which are known beneficial microbial taxa such as *Lachnospiraceae*, *Dorea*, *Oscillospira*, and *Ruminococcus* (Fig 4A). Modularity is thought to enhance the adaptability of the microbiome through enhanced horizontal gene transfer between species[32] or by promoting the acquisition of novel functions by making the overall metabolic network more robust to the addition of novel functions[33, 34]. Modularity has been positively correlated with environmental variability. For commensal organisms in the gut, host factors as well as other bacteria or their metabolites can provide the necessary environmental variation to drive module formation. Given the highly controlled nature of the environment and diet these mice experienced, we believe variable host factors, and in particular antibodies) may facilitate module formation. Higher connectance and/or modularity is also thought to increase resistance of networks to biological invasion, which can be extrapolated to include pathogen colonization of the gut. These emergent properties of the microbial network could make MHC heterozygotes more resistant to enteric infection, which has been demonstrated previously in MHC heterozygous mice infected with *Salmonella*[1, 17, 18]. Finally, less connected and less modular microbiomes have been associated with disease states in humans including obesity, inflammatory bowel disease, and alcoholism[35–37].

Multiple gene functions were differentially abundant between MHC homozygotes and MHC heterozygotes. In an effort to describe how the effect of MHC heterozygosity on microbiome form and function might impact host fitness, we have focused our discussion on those genes whose functions are known to be important for host health. Carbohydrate metabolism is an essential function provided by members of the microbiota that is key for nutrient extraction from complex polysaccharides derived from plant tissues[38, 39]. However, the mucus lining of the gut epithelium is also covered in host-derived carbohydrates in the form of glycan, and this glycan layer is an important element of barrier defense in the gut that limits inflammation[40–43]. Multiple genes encoding enzymes that degrade host-derived glycans were enriched in MHC homozygotes compared to MHC heterozygotes (Fig 5). Microbial digestion of host-derived carbohydrates that comprise the mucus layer in the gut have recently been shown to enhance intestinal permeability and susceptibility to enteric infection in mice[44], and mucus-deficient mice develop spontaneous colitis[40]. Thus, barrier integrity could be reduced in MHC homozygotes compared to MHC heterozygotes because of reduced thickness of the mucus lining of the gut, and this may reduce fitness by pre-disposing individuals to acute (e.g. septicemia) and chronic (e.g. liver dysfunction) diseases. Additionally, MHC homozygotes have higher representation of predicted genes involved in the degradation of other glycans such as dietary fibers including N4-(beta-N-acetylglucosaminyl)-L-asparaginase [EC:3.5.1.26], and levanase [EC:3.2.1.65]. This observation probably explains why their gut microbiomes have higher abundance of genes involved in metabolism of other carbohydrates such as cellulose (cellulose synthase (UDP-forming) [EC:2.4.1.12]), sucrose (trehalose-6-phosphate hydrolase [EC:3.2.1.93]), and galactose (galactonate dehydratase [EC:4.2.1.6]). Higher potential access to carbohydrates from diet and host may favor a specific group of microorganisms in the colon with the ability to perform D-lactate fermentation, explaining why MHC homozygote microbiomes have increased D-lactate dehydrogenase gene. Increased accumulation of D-lactate in the blood causes D-lactate acidosis that can promote neurological disease and is linked to a gastrointestinal disorder called short-bowel syndrome[45, 46]. These data also imply that MHC homozygotes may be more susceptible to diseases associated with diets high in sugar like diabetes and colitis, which has been reported before in MHC congenic mouse models as well as humans[47–51].

Numerous bacterial metabolites are utilized by gut epithelial cells to enhance barrier integrity. Commensal-derived essential amino acids (EAAs) (e.g. tryptophan) have recently been shown to promote barrier integrity by upregulating IL-22 expression in gut epithelial cells[52]. In addition to the genes important for the biosynthesis of these EAAs, heterozygote microbiomes are enriched for genes associated with polyamine production (carboxynorspermidine decarboxylase [EC:4.1.1.-], putrescine carbamoyltransferase [EC:2.1.3.6]). Polyamines such as spermidine, putrescine are some of the most important molecules produced by the microbiota that are essential in regulating not only barrier integrity[53, 54], but also anti-inflammation, anti-mutagenicity, and autophagy[55]. In contrast, homozygotes are enriched in genes important for the synthesis of compounds known to decrease barrier integrity. For example, alcohol dehydrogenase (NADP+) [EC 1.1.1.71] catalyzes the conversion of ethanol to acetaldehydes, which disrupts the mucosal barrier in the intestine[56]. In addition, homozygote microbiomes have higher abundance of genes associated with the production of bacterial toxins (exfoliative toxin A/B) that might damage epithelial cells, and bacterial motility (chemotaxis protein MotB). Together, these observations suggest that MHC homozygotes may have a gut barrier deficiency that may be more permissive to leakage of pro-inflammatory microbial metabolites or whole bacteria from the gut into deeper host tissues.

MHC heterozygotes were enriched for genes whose functions are known to be important for dietary energy harvest and biosynthesis of essential nutrients. Indeed, twice as many genes associated with energy metabolism and amino acid synthesis were enriched in MHC heterozygotes compared to MHC homozygotes. MHC heterozygotes have higher abundance of microbial genes associated with terpenoid (also known as isoprenoid) biosynthesis via the methylerythritol phosphate (MEP) pathway (1-deoxy-D-xylulose-5-phosphate synthase [EC:2.2.1.7], geranylgeranyl diphosphate synthase, type II [EC:2.5.1.1 2.5.1.10 2.5.1.29]), which is important for the biosynthesis of vitamins essential for host health, including B1 (thiamine) and B6 (pyridoxine)[57]. The TCA cycle is an essential component of cellular respiration process through the oxidation of acetyl-CoA, with central importance in energy harvest, and biosynthesis of essential nutrients including amino acid, SCFAs. SCFAs are entirely produced by the microbiota[58, 59], which are particularly important for enterocyte health[60, 61], and have anti-carcinogenic and anti-inflammatory properties[62]. In addition, the microbiomes of MHC heterozygotes were enriched for genes that provide substrate for the TCA cycle (pyruvate ferredoxin oxidoreductase, beta subunit [EC:1.2.7.1]) as well as enzymes directly associated with the TCA pathway (succinate dehydrogenase flavoprotein subunit [EC:1.3.99.1], fumarate reductase flavoprotein subunit [EC:1.3.99.1] and malate dehydrogenase (oxaloacetate-decarboxylating)(NADP+) [EC:1.1.1.40]). Another striking difference between MHC homozygote and MHC heterozygote core microbiomes was the differential abundance of genes associated with methane metabolism (Fig 6). While MHC homozygote microbiomes had higher gene abundances associated with the synthesis of methane, heterozygote microbiomes had higher gene abundances for the utilization of it. KEGG pathways analysis showed that methanogens in the homozygotes employ reductive acetyl-CoA pathway in methanogenesis, whereas the methanotrophic pathway in MHC heterozygote contributes to the synthesis of acetyl-CoA and pyruvate. Production of methane is a major pathway for removing fermentation products including H_2_ from gut ecosystem[63], which may enhance fermentation efficiency such as D-lactate synthesis. In addition, previous studies showed the links between methanogenesis and a pathological increase of bacteria in the small intestine known as small intestinal bacterial overgrowth (SIBO)[64], which may contribute to irritable bowel syndrome (IBS)[65]. Two other important genes enriched in MHC heterozygotes are enoyl-CoA hydratase [EC:4.2.1.17], which catalyzes the synthesis of acetyl-CoA (and energy) from fatty acids, and acetyl-CoA carboxylase [EC:6.4.1.2], which catalyzes the conversion of acetyl-CoA into malonyl-CoA for fatty acid synthesis. While acetyl-CoA can be funneled into TCA cycles, malonyl-CoA is a precursor of fatty acid biosynthesis, suggesting that MHC heterozygote microbiomes may maintain a better tradeoff in fatty acid metabolism critical for host health.

Finally, Commensal microbes are important for the detoxification of xenobiotic compounds[66, 67], which could impact the host health in a variety of ways. The fecal microbiomes of MHC heterozygotes are enriched for genes whose functions are important for the detoxification of respiration-associated reactive oxygen species such as catalase, chloride peroxidase [EC:1.11.1.10], glutathione production (6-phosphogluconate dehydrogenase [EC:1.1.1.44], glucose-6-phosphate 1-dehydrogenase [EC:1.1.1.49]. These enzymes are important for scavenging/neutralizing free radicals, peroxides, and lipid peroxides formed during cellular respiration, suggesting that MHC heterozygotes may be less susceptible to diseases associated with oxidative stress.

It is important to acknowledge major caveats to the observations we describe in this study. The first stem from technological limitations of predictive meta-genomic approaches based on 16S rRNA sequence data. Many of the operational taxonomic units (OTUs) identified from gut ecosystem remain undefined based on our current knowledge. When defined, the biology of many of them is not fully understood, which challenges us to explore and compare the complete functional roles between any two systems. As a consequence of this, we cannot rule out that an observed functional advantage contributed by a microbial species found in heterozygotes cannot be rescued by a currently undefined microbial species found in MHC homozygotes. Additionally, PICRUSt annotated metabolic pathways are based on the KEGG reference database. This database contains all currently known microbial functions, but is only a small subset of the total possible functions (based on coding sequence). Therefore, many physiologically important functions will go unappreciated based on this analysis method. Also, because PICRUSt is based on 16S rRNA genes, eukaryotic microbial and viral contributions are ignored in this analysis. Finally, it is not within the scope of this manuscript to compare/contrast all of the unique features of the microbiota/microbiomes observed among our six genotypes. To simplify the story we have focused on core (i.e. shared) species and functions among MHC homozygotes and MHC heterozygotes and it must be explicitly restated that non-core species excluded from our analyses will contribute many novel functions to the microbiome that we do not have the resolution to discuss here. Finally, as stated above, more MHC homozygote and MHC heterozygote comparisons are needed to assess the generality of these effects. Given these limitations, we believe this study provides a framework for the generation of novel hypotheses regarding the influence of MHC-microbiota interactions on host physiology.

Given the role MHCII antigen presentation plays in the generation of antibody responses, we believe that MHC polymorphisms differentially regulate anti-commensal antibody responses to drive differences in community structure/function. If true, then MHC-mediated deficiencies in this response could result in unstable microbiomes that can enhance susceptibility microbiota-dependent diseases. Indeed, genes within the MHC, and MHCII specifically, have recently been strongly linked to IgA deficiency in humans[68–71] and IgA deficiency has been associated with increased susceptibility to a variety of autoimmune, inflammatory, and infectious diseases in humans. Additionally, it has also been shown that MHCII expression by group 3 innate lymphoid cells is important for inducing tolerance against commensal antigens[3, 72], and that non-classical MHC class I-like genes have been identified that can present unconventional antigens derived from the microbiota to influence immune development and microbiota composition. For example, a recent study has demonstrated that a non-classical MHC class I molecule (CD1d), that presents lipid antigens and is central to development and activation of natural killer-like T (NKT) cells in the gut[73], may also be important for sculpting microbiota composition[74]. Another non-classical MHC class I-like molecule, MR1, has been shown to bind and present B vitamins, which are exclusively produced by the microbiota, to drive the activation of mucosa-associated invariant T (MAIT) cells[75]. Thus, accumulating evidence suggests that classical MHC genes can operate through unconventional means to influence the fitness-consequences of host-microbiota interactions, and that non-classical MHC-like molecules represent novel mechanisms facilitating molecular crosstalk between the microbiota and various aspects of the mucosal immune system.

MHC polymorphisms can be favored evolutionarily if they promote the establishment of a beneficial microbiome. The benefits of our microbiomes arise primarily from the metabolic services and protection from infection they provide us. Results from our experiments provide support for the hypothesis that MHC heterozygosity is favored because it positively influences microbiome functionality. Future studies directly testing the physiologic predictions arising from our results are underway. Multiple studies in humans have revealed links between the presence of specific HLA alleles and susceptibility to autoimmune, infectious, and inflammatory diseases including rheumatoid arthritis[76], multiple sclerosis[77], inflammatory bowel disease[78–81], lupus[82, 83], and diabetes[84], in which the microbiome has been implicated as a contributing factor[85–87]. To what degree (and how) an individual’s MHC genotype contributes to molding the community of symbiotic microbes and how this influences host health are not understood. Collectively, our data complement the studies demonstrating that MHC polymorphisms can influence microbiota composition. However, we also make an effort to describe the impact of MHC heterozygosity on microbiome function, and provide preliminary evidence to support the hypothesis that MHC heterozygosity may be evolutionarily favored by promoting the establishment of a more beneficial microbiome. Given the life-long association between vertebrate hosts and their microbiota and the pervasive impact these symbionts have on host physiology, these interactions may have been important drivers of MHC gene evolution. In conclusion, we make no allusion that the data generated from six genotypes of inbred mice (maintained in ventilated cages, eating the same irradiated chow, housed under specific-pathogen-free conditions, etc) reflects a natural condition. The true biological relevance of such interactions need to be studied by Evolutionary Ecologists in the field. Excitingly, this has already begun. It is our hope that this descriptive/speculative study provokes conversation within such groups on the possibility that MHC polymorphism may regulate fitness through modulation of the microbiome.

## Supporting information

S1 Fig MHC congenic BALB/c mice were purchased from Jackson Laboratories representing three different MHC genotypes (H2^*b/b*^, H2^*d/d*^, and H2^*k/k*^ MHC genotypes). These animals were used as founders to re-derive MHC homozygote and MHC heterozygote animals for use in this study.
(TIFF)

S2 Fig **A)** PCoA of beta-diversity comparison of overall community between MHC homozygote and MHC heterozygote animals based on Unweighted Unifrac analysis (ANOSIM: *R* = 0.18; *P* = 0.01; no. of permutations = 99). **B)** PCoA of beta-diversity comparison of core community between MHC homozygote and MHC heterozygote animals based on Weighted Unifrac analysis (ANOSIM: *R* = 0.06; *P* > 0.05; no. of permutations = 99).
(TIFF)

S1 Table Differential enrichment of specific taxa within the overall microbiota of MHC homozygote and MHC heterozygote animals, which are represented as log_2_FoldChange values with *P* < 0.05, Benjamini-Hochberg adjusted. The positive log_2_FoldChange values indicate that the taxa enriched in MHC homozygotes, while negative values indicate the taxa enriched in MHC heterozygotes.
(DOCX)

S2 Table Distribution of betweenness centrality values of nodes with their closed relatives across genotype-level networks. If an OTU is not calculated as a node in a network, its value is reported as N/A.
(XLSX)

S3 Table Summary of the weighted Nearest Sequenced Taxon Index (weighted NSTI) scores of each of the samples used in this study. The score represents the extent to which OTUs in a given sample are related to their sequenced genomes.
(DOCX)

S4 Table Differential enrichment of predicted KO genes involved in the KEGG metabolic pathways within the core microbiota of MHC homozygote and MHC heterozygote animals, which are represented as log_2_FoldChange values with *P* < 0.05, Benjamini-Hochberg adjusted. The positive log_2_FoldChange values indicate that the taxa are enriched in MHC homozygotes, while negative values indicate that the taxa are enriched in MHC heterozygotes.
(XLSX)

## References

[1] Kubinak JL, Stephens WZ, Soto R, Petersen C, Chiaro T, Gogokhia L, et al MHC variation sculpts individualized microbial communities that control susceptibility to enteric infection. Nat Commun. 2015;6:8642 10.1038/ncomms9642 . 26494419 PMC4621775

[2] Oliphant CJ, Hwang YY, Walker JA, Salimi M, Wong SH, Brewer JM, et al MHCII-mediated dialog between group 2 innate lymphoid cells and CD4(+) T cells potentiates type 2 immunity and promotes parasitic helminth expulsion. Immunity. 2014;41(2):283–95. 10.1016/j.immuni.2014.06.016 . 25088770 PMC4148706

[3] Hepworth MR, Monticelli LA, Fung TC, Ziegler CG, Grunberg S, Sinha R, et al Innate lymphoid cells regulate CD4+ T-cell responses to intestinal commensal bacteria. Nature. 2013;498(7452):113–7. 10.1038/nature12240 . 23698371 PMC3699860

[4] Haylett RS, Koch N, Rink L. MHC class II molecules activate NFAT and the ERK group of MAPK through distinct signaling pathways in B cells. European journal of immunology. 2009;39(7):1947–55. 10.1002/eji.200838992 . 19544309

[5] St-Pierre Y, Nabavi N, Ghogawala Z, Glimcher LH, Watts TH. A functional role for signal transduction via the cytoplasmic domains of MHC class II proteins. Journal of immunology. 1989;143(3):808–12. . 2473111

[6] Newell MK, VanderWall J, Beard KS, Freed JH. Ligation of major histocompatibility complex class II molecules mediates apoptotic cell death in resting B lymphocytes. Proc Natl Acad Sci U S A. 1993;90(22):10459–63. 10.1073/pnas.90.22.10459 . 8248132 PMC47796

[7] Tiwari JL, Terasaki PI. HLA and disease associations. 1 ed New York: Springer-Verlag; 1985.

[8] Apanius V, Penn D, Slev P, Ruff LR, Potts WK. The nature of selection on the major histocompatibility complex. Critical Reviews in Immunology. 1997;17:179–224. 9094452 10.1615/critrevimmunol.v17.i2.40

[9] Cowell LG, Kepler TB, Janitz M, Lauster R, Mitchison NA. The distribution of variation in regulatory gene segments, as present in MHC class II promoters. Genome research. 1998;8(2):124–34. . 9477340 10.1101/gr.8.2.124

[10] Janitz M, Mitchison A, Reiners-Schramm L, Lauster R. Polymorphic MHC class II promoters exhibit distinct expression pattern in various antigen-presenting cell lines. Tissue antigens. 1997;49(2):99–106. . 9062963 10.1111/j.1399-0039.1997.tb02721.x

[11] Potts WK, Slev PR. Pathogen-based models favoring MHC genetic diversity. Immunological Reviews. 1995;143:181–97. 7558076 10.1111/j.1600-065x.1995.tb00675.x

[12] Penn D, Potts W. The evolution of mating preferences and major histocompatibility genes. American Naturalist. 1999;153:145–64. 10.1086/303166 29578757

[13] Thursz MR, Thomas HC, Greenwood BM, Hill AV. Heterozygote advantage for HLA class-II type in hepatitis B virus infection. Nat Genet. 1997;17(1):11–2. 10.1038/ng0997-11 . 9288086

[14] Carrington M, Nelson GW, Martin MP, Kissner T, Vlahov D, Goedert JJ, et al HLA and HIV-1: heterozygote advantage and B*35-Cw*04 disadvantage. Science. 1999;283(5408):1748–52. . 10073943 10.1126/science.283.5408.1748

[15] Tang J, Costello C, Keet IP, Rivers C, Leblanc S, Karita E, et al HLA class I homozygosity accelerates disease progression in human immunodeficiency virus type 1 infection. AIDS Res Hum Retroviruses. 1999;15(4):317–24. 10.1089/088922299311277 . 10082114

[16] Jeffery KJ, Siddiqui AA, Bunce M, Lloyd AL, Vine AM, Witkover AD, et al The influence of HLA class I alleles and heterozygosity on the outcome of human T cell lymphotropic virus type I infection. Journal of immunology. 2000;165(12):7278–84. . 11120862 10.4049/jimmunol.165.12.7278

[17] Penn DJ, Damjanovich K, Potts WK. MHC heterozygosity confers a selective advantage against multiple-strain infections. Proc Natl Acad Sci U S A. 2002;99(17):11260–4. 10.1073/pnas.162006499 . 12177415 PMC123244

[18] McClelland EE, Penn DJ, Potts WK. Major histocompatibility complex heterozygote superiority during coinfection. Infection and immunity. 2003;71(4):2079–86. 10.1128/IAI.71.4.2079-2086.2003 . 12654829 PMC152037

[19] Oliver MK, Telfer S, Piertney SB. Major histocompatibility complex (MHC) heterozygote superiority to natural multi-parasite infections in the water vole (Arvicola terrestris). Proc Biol Sci. 2009;276(1659):1119–28. 10.1098/rspb.2008.1525 . 19129114 PMC2679068

[20] Froeschke G, Sommer S. MHC class II DRB variability and parasite load in the striped mouse (Rhabdomys pumilio) in the Southern Kalahari. Mol Biol Evol. 2005;22(5):1254–9. 10.1093/molbev/msi112 . 15703235

[21] Evans ML, Neff BD. Major histocompatibility complex heterozygote advantage and widespread bacterial infections in populations of Chinook salmon (Oncorhynchus tshawytscha). Molecular ecology. 2009;18(22):4716–29. 10.1111/j.1365-294X.2009.04374.x . 19821902

[22] Ortego J, Aparicio JM, Calabuig G, Cordero PJ. Risk of ectoparasitism and genetic diversity in a wild lesser kestrel population. Molecular ecology. 2007;16(17):3712–20. 10.1111/j.1365-294X.2007.03406.x . 17845443

[23] Nevo E, Beiles A. Selection for class II Mhc heterozygosity by parasites in subterranean mole rats. Experientia. 1992;48(5):512–5. . 1351001 10.1007/BF01928177

[24] Sommer F, Backhed F. The gut microbiota—masters of host development and physiology. Nature reviews Microbiology. 2013;11(4):227–38. 10.1038/nrmicro2974 23435359

[25] Buffie CG, Pamer EG. Microbiota-mediated colonization resistance against intestinal pathogens. Nature reviews Immunology. 2013;13(11):790–801. 10.1038/nri3535 . 24096337 PMC4194195

[26] Toivanen P, Vaahtovuo J, Eerola E. Influence of major histocompatibility complex on bacterial composition of fecal flora. Infection and immunity. 2001;69(4):2372–7. 10.1128/IAI.69.4.2372-2377.2001 . 11254595 PMC98167

[27] Vaahtovuo J, Toivanen P, Eerola E. Study of murine faecal microflora by cellular fatty acid analysis; effect of age and mouse strain. Antonie van Leeuwenhoek. 2001;80(1):35–42. . 11761365 10.1023/a:1012058107731

[28] Bolnick DI, Snowberg LK, Gregory Caporaso J, Lauber C, Knight R, Stutz WE. Major Histocompatibility Complex class II polymorphism influences gut microbiota composition and diversity. Molecular ecology. 2014 10.1111/mec.12846 24975397

[29] Markle JG, Frank DN, Mortin-Toth S, Robertson CE, Feazel LM, Rolle-Kampczyk U, et al Sex differences in the gut microbiome drive hormone-dependent regulation of autoimmunity. Science. 2013;339(6123):1084–8. 10.1126/science.1233521 . 23328391

[30] Muegge BD, Kuczynski J, Knights D, Clemente JC, Gonzalez A, Fontana L, et al Diet drives convergence in gut microbiome functions across mammalian phylogeny and within humans. Science. 2011;332(6032):970–4. 10.1126/science.1198719 . 21596990 PMC3303602

[31] Moya A, Ferrer M. Functional Redundancy-Induced Stability of Gut Microbiota Subjected to Disturbance. Trends in microbiology. 2016;24(5):402–13. 10.1016/j.tim.2016.02.002 . 26996765

[32] Kreimer A, Borenstein E, Gophna U, Ruppin E. The evolution of modularity in bacterial metabolic networks. Proc Natl Acad Sci U S A. 2008;105(19):6976–81. 10.1073/pnas.0712149105 . 18460604 PMC2383979

[33] Wilke CO, Adami C. Evolution of mutational robustness. Mutat Res. 2003;522(1–2):3–11. . 12517406 10.1016/s0027-5107(02)00307-x

[34] Wagner A. Robustness and evolvability: a paradox resolved. Proc Biol Sci. 2008;275(1630):91–100. 10.1098/rspb.2007.1137 . 17971325 PMC2562401

[35] Greenblum S, Turnbaugh PJ, Borenstein E. Metagenomic systems biology of the human gut microbiome reveals topological shifts associated with obesity and inflammatory bowel disease. Proc Natl Acad Sci U S A. 2012;109(2):594–9. 10.1073/pnas.1116053109 . 22184244 PMC3258644

[36] Naqvi A, Rangwala H, Keshavarzian A, Gillevet P. Network-based modeling of the human gut microbiome. Chem Biodivers. 2010;7(5):1040–50. 10.1002/cbdv.200900324 . 20491063 PMC3681515

[37] Baldassano SN, Bassett DS. Topological distortion and reorganized modular structure of gut microbial co-occurrence networks in inflammatory bowel disease. Sci Rep. 2016;6:26087 10.1038/srep26087 . 27188829 PMC4870640

[38] Hooper LV, Midtvedt T, Gordon JI. How host-microbial interactions shape the nutrient environment of the mammalian intestine. Annual Reviews of Nutrition. 2002;22:283–307. 10.1146/annurev.nutr.22.011602.092259 12055347

[39] Rowland I, Gibson G, Heinken A, Scott K, Swann J, Thiele I, et al Gut microbiota functions: metabolism of nutrients and other food components. Eur J Nutr. 2017 10.1007/s00394-017-1445-8 . 28393285 PMC5847071

[40] Van der Sluis M, De Koning BA, De Bruijn AC, Velcich A, Meijerink JP, Van Goudoever JB, et al Muc2-deficient mice spontaneously develop colitis, indicating that MUC2 is critical for colonic protection. Gastroenterology. 2006;131(1):117–29. 10.1053/j.gastro.2006.04.020 16831596

[41] Johansson ME, Gustafsson JK, Sjoberg KE, Petersson J, Holm L, Sjovall H, et al Bacteria penetrate the inner mucus layer before inflammation in the dextran sulfate colitis model. PloS one. 2010;5(8):e12238 10.1371/journal.pone.0012238 . 20805871 PMC2923597

[42] Johansson ME, Phillipson M, Petersson J, Velcich A, Holm L, Hansson GC. The inner of the two Muc2 mucin-dependent mucus layers in colon is devoid of bacteria. Proc Natl Acad Sci U S A. 2008;105(39):15064–9. 10.1073/pnas.0803124105 . 18806221 PMC2567493

[43] Hansson GC. Role of mucus layers in gut infection and inflammation. Current opinion in microbiology. 2012;15(1):57–62. 10.1016/j.mib.2011.11.002 . 22177113 PMC3716454

[44] Desai MS, Seekatz AM, Koropatkin NM, Kamada N, Hickey CA, Wolter M, et al A Dietary Fiber-Deprived Gut Microbiota Degrades the Colonic Mucus Barrier and Enhances Pathogen Susceptibility. Cell. 2016;167(5):1339–53 e21. 10.1016/j.cell.2016.10.043 . 27863247 PMC5131798

[45] Petersen C. D-lactic acidosis. Nutr Clin Pract. 2005;20(6):634–45. 10.1177/0115426505020006634 . 16306301

[46] Kowlgi NG, Chhabra L. D-lactic acidosis: an underrecognized complication of short bowel syndrome. Gastroenterol Res Pract. 2015;2015:476215 10.1155/2015/476215 . 25977687 PMC4421027

[47] Wu AY, Schulman SJ, Marconi LA, Reilly CR, Scott B, Lo D. Protection against diabetes by MHC heterozygosity and reversal by cyclophosphamide. Cellular immunology. 1998;184(2):112–20. 10.1006/cimm.1998.1269 . 9630837

[48] Goyette P, Boucher G, Mallon D, Ellinghaus E, Jostins L, Huang H, et al High-density mapping of the MHC identifies a shared role for HLA-DRB1*01:03 in inflammatory bowel diseases and heterozygous advantage in ulcerative colitis. Nat Genet. 2015;47(2):172–9. 10.1038/ng.3176 . 25559196 PMC4310771

[49] Matzaraki V, Kumar V, Wijmenga C, Zhernakova A. The MHC locus and genetic susceptibility to autoimmune and infectious diseases. Genome Biology. 2017;18(1):76 10.1186/s13059-017-1207-1 28449694 PMC5406920

[50] Wicker LS, Miller BJ, Coker LZ, McNally SE, Scott S, Mullen Y, et al Genetic control of diabetes and insulitis in the nonobese diabetic (NOD) mouse. J Exp Med. 1987;165(6):1639–54. 10.1084/jem.165.6.1639 . 3585250 PMC2188363

[51] Ruff JS, Suchy AK, Hugentobler SA, Sosa MM, Schwartz BL, Morrison LC, et al Human-relevant levels of added sugar consumption increase female mortality and lower male fitness in mice. Nat Commun. 2013;4:2245 10.1038/ncomms3245 . 23941916 PMC3775329

[52] Zelante T, Iannitti RG, Cunha C, De Luca A, Giovannini G, Pieraccini G, et al Tryptophan catabolites from microbiota engage aryl hydrocarbon receptor and balance mucosal reactivity via interleukin-22. Immunity. 2013;39(2):372–85. 10.1016/j.immuni.2013.08.003 . 23973224

[53] Lux GD, Marton LJ, Baylin SB. Ornithine decarboxylase is important in intestinal mucosal maturation and recovery from injury in rats. Science. 1980;210(4466):195–8. . 6774420 10.1126/science.6774420

[54] Loser C, Eisel A, Harms D, Folsch UR. Dietary polyamines are essential luminal growth factors for small intestinal and colonic mucosal growth and development. Gut. 1999;44(1):12–6. 10.1136/gut.44.1.12 . 9862820 PMC1760068

[55] Matsumoto M, Kurihara S, Kibe R, Ashida H, Benno Y. Longevity in mice is promoted by probiotic-induced suppression of colonic senescence dependent on upregulation of gut bacterial polyamine production. PloS one. 2011;6(8):e23652 10.1371/journal.pone.0023652 . 21858192 PMC3156754

[56] Chen P, Schnabl B. Host-microbiome interactions in alcoholic liver disease. Gut Liver. 2014;8(3):237–41. 10.5009/gnl.2014.8.3.237 . 24827618 PMC4026639

[57] Rodriguez-Concepcion M, Boronat A. Elucidation of the methylerythritol phosphate pathway for isoprenoid biosynthesis in bacteria and plastids. A metabolic milestone achieved through genomics. Plant Physiol. 2002;130(3):1079–89. 10.1104/pp.007138 . 12427975 PMC1540259

[58] Miller TL, Wolin MJ. Pathways of acetate, propionate, and butyrate formation by the human fecal microbial flora. Applied and environmental microbiology. 1996;62(5):1589–92. . 8633856 10.1128/aem.62.5.1589-1592.1996 PMC167932

[59] Flint HJ, Duncan SH, Scott KP, Louis P. Links between diet, gut microbiota. composition and gut metabolism. Proceedings of the Nutritional Society. 2015;74:13–22. 10.1017/S0029665114001463 25268552

[60] Andoh A, Tsujikawa T, Fujiyama Y. Role of dietary fiber and short-chain fatty acids in the colon. Curr Pharm Des. 2003;9(4):347–58. . 12570825 10.2174/1381612033391973

[61] Scheppach W. Effects of short chain fatty acids on gut morphology and function. Gut. 1994;35(1 Suppl):S35–8. 10.1136/gut.35.1_suppl.s35 . 8125387 PMC1378144

[62] Greer JB, O’Keefe SJ. Microbial induction of immunity, inflammation, and cancer. Front Physiol. 2011;1:168 10.3389/fphys.2010.00168 . 21423403 PMC3059938

[63] Stams AJ. Metabolic interactions between anaerobic bacteria in methanogenic environments. Antonie van Leeuwenhoek. 1994;66(1–3):271–94. . 7747937 10.1007/BF00871644

[64] Attaluri A, Jackson M, Valestin J, Rao SS. Methanogenic flora is associated with altered colonic transit but not stool characteristics in constipation without IBS. Am J Gastroenterol. 2010;105(6):1407–11. 10.1038/ajg.2009.655 . 19953090 PMC3822765

[65] Shah ED, Basseri RJ, Chong K, Pimentel M. Abnormal breath testing in IBS: a meta-analysis. Digestive diseases and sciences. 2010;55(9):2441–9. 10.1007/s10620-010-1276-4 . 20467896

[66] Koppel N, Maini Rekdal V, Balskus EP. Chemical transformation of xenobiotics by the human gut microbiota. Science. 2017;356(6344). 10.1126/science.aag2770 . 28642381 PMC5534341

[67] Kohl KD, Dearing MD. The Woodrat Gut Microbiota as an Experimental System for Understanding Microbial Metabolism of Dietary Toxins. Frontiers in microbiology. 2016;7:1165 10.3389/fmicb.2016.01165 . 27516760 PMC4963388

[68] Ferreira RC, Pan-Hammarstrom Q, Graham RR, Fontan G, Lee AT, Ortmann W, et al High-density SNP mapping of the HLA region identifies multiple independent susceptibility loci associated with selective IgA deficiency. PLoS genetics. 2012;8(1):e1002476 10.1371/journal.pgen.1002476 . 22291608 PMC3266887

[69] Kralovicova J, Hammarstrom L, Plebani A, Webster AD, Vorechovsky I. Fine-scale mapping at IGAD1 and genome-wide genetic linkage analysis implicate HLA-DQ/DR as a major susceptibility locus in selective IgA deficiency and common variable immunodeficiency. Journal of immunology. 2003;170(5):2765–75. . 12594308 10.4049/jimmunol.170.5.2765

[70] Vorechovsky I, Webster AD, Plebani A, Hammarstrom L. Genetic linkage of IgA deficiency to the major histocompatibility complex: evidence for allele segregation distortion, parent-of-origin penetrance differences, and the role of anti-IgA antibodies in disease predisposition. Am J Hum Genet. 1999;64(4):1096–109. . 10090895 10.1086/302326 PMC1377834

[71] Volanakis JE, Zhu ZB, Schaffer FM, Macon KJ, Palermos J, Barger BO, et al Major histocompatibility complex class III genes and susceptibility to immunoglobulin A deficiency and common variable immunodeficiency. The Journal of clinical investigation. 1992;89(6):1914–22. 10.1172/JCI115797 . 1351062 PMC295891

[72] Hepworth MR, Fung TC, Masur SH, Kelsen JR, McConnell FM, Dubrot J, et al Immune tolerance. Group 3 innate lymphoid cells mediate intestinal selection of commensal bacteria-specific CD4(+) T cells. Science. 2015;348(6238):1031–5. 25908663 10.1126/science.aaa4812 PMC4449822

[73] Girardi E, Zajonc DM. Molecular basis of lipid antigen presentation by CD1d and recognition by natural killer T cells. Immunol Rev. 2012;250(1):167–79. 10.1111/j.1600-065X.2012.01166.x . 23046129 PMC3471380

[74] de Guinoa JS, Jimeno R, Gaya M, Kipling D, Garzon MJ, Dunn-Walters DK, et al CD1d-mediated lipid presentation by CD11c+ cells regulates intestinal homeostasis. EMBO. 2018;37(5):1–17. 10.15252/embj.201797537 PMC5830915 29378774

[75] Kjer-Nielsen L, Patel O, Corbett AJ, Le Nours J, Meehan B, Liu L, et al MR1 presents microbial vitamin B metabolites to MAIT cells. Nature. 2012;491(7426):717–23. 10.1038/nature11605 . 23051753

[76] Deighton CM, Walker DJ, Griffiths ID, Roberts DF. The contribution of HLA to rheumatoid arthritis. Clin Genet. 1989;36(3):178–82. . 2676268 10.1111/j.1399-0004.1989.tb03185.x

[77] Harbo HF, Lie BA, Sawcer S, Celius EG, Dai KZ, Oturai A, et al Genes in the HLA class I region may contribute to the HLA class II-associated genetic susceptibility to multiple sclerosis. Tissue antigens. 2004;63(3):237–47. 10.1111/j.0001-2815.2004.00173.x . 14989713

[78] Hampe J, Schreiber S, Shaw SH, Lau KF, Bridger S, Macpherson AJ, et al A genomewide analysis provides evidence for novel linkages in inflammatory bowel disease in a large European cohort. Am J Hum Genet. 1999;64(3):808–16. 10.1086/302294 . 10053016 PMC1377799

[79] Satsangi J, Parkes M, Louis E, Hashimoto L, Kato N, Welsh K, et al Two stage genome-wide search in inflammatory bowel disease provides evidence for susceptibility loci on chromosomes 3, 7 and 12. Nat Genet. 1996;14(2):199–202. 10.1038/ng1096-199 . 8841195

[80] Satsangi J, Welsh KI, Bunce M, Julier C, Farrant JM, Bell JI, et al Contribution of genes of the major histocompatibility complex to susceptibility and disease phenotype in inflammatory bowel disease. Lancet. 1996;347(9010):1212–7. . 8622450 10.1016/s0140-6736(96)90734-5

[81] Cho JH, Nicolae DL, Gold LH, Fields CT, LaBuda MC, Rohal PM, et al Identification of novel susceptibility loci for inflammatory bowel disease on chromosomes 1p, 3q, and 4q: evidence for epistasis between 1p and IBD1. Proc Natl Acad Sci U S A. 1998;95(13):7502–7. 10.1073/pnas.95.13.7502 . 9636179 PMC22666

[82] Forabosco P, Gorman JD, Cleveland C, Kelly JA, Fisher SA, Ortmann WA, et al Meta-analysis of genome-wide linkage studies of systemic lupus erythematosus. Genes Immun. 2006;7(7):609–14. 10.1038/sj.gene.6364338 . 16971955

[83] Cortes LM, Baltazar LM, Lopez-Cardona MG, Olivares N, Ramos C, Salazar M, et al HLA class II haplotypes in Mexican systemic lupus erythematosus patients. Human immunology. 2004;65(12):1469–76. 10.1016/j.humimm.2004.09.008 . 15603875

[84] Todd JA. Genetic control of autoimmunity in type 1 diabetes. Immunology today. 1990;11(4):122–9. . 2187469 10.1016/0167-5699(90)90049-f

[85] Chervonsky AV. Microbiota and autoimmunity. Cold Spring Harb Perspect Biol. 2013;5(3):a007294 10.1101/cshperspect.a007294 . 23457255 PMC3578358

[86] Bhargava P, Mowry EM. Gut microbiome and multiple sclerosis. Curr Neurol Neurosci Rep. 2014;14(10):492 10.1007/s11910-014-0492-2 . 25204849

[87] Sokol H, Seksik P, Rigottier-Gois L, Lay C, Lepage P, Podglajen I, et al Specificities of the fecal microbiota in inflammatory bowel disease. Inflammatory bowel diseases. 2006;12(2):106–11. 10.1097/01.MIB.0000200323.38139.c6 . 16432374

