# Supplementary figures and images for: Does MHC heterozygosity influence microbiota form and function?
Source: PLoS One. 2019 May 16;14(5):e0215946. doi: 10.1371/journal.pone.0215946 (PMC6522005; doi:10.1371/journal.pone.0215946)

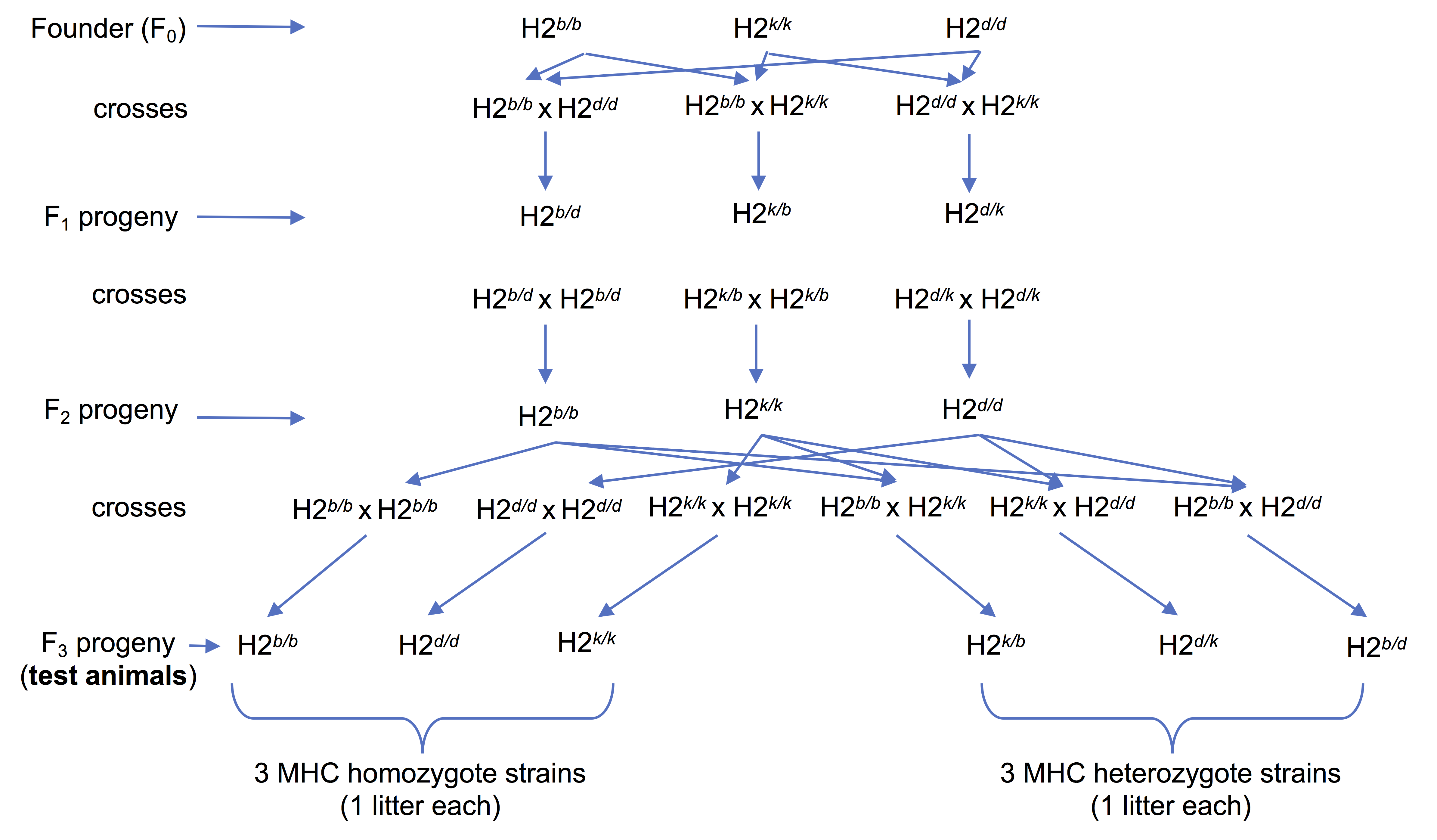

Supplement: S1 Fig — These animals were used as founders to re-derive MHC homozygote and MHC heterozygote animals for use in this study. (TIFF) [file pone.0215946.s001.tiff]

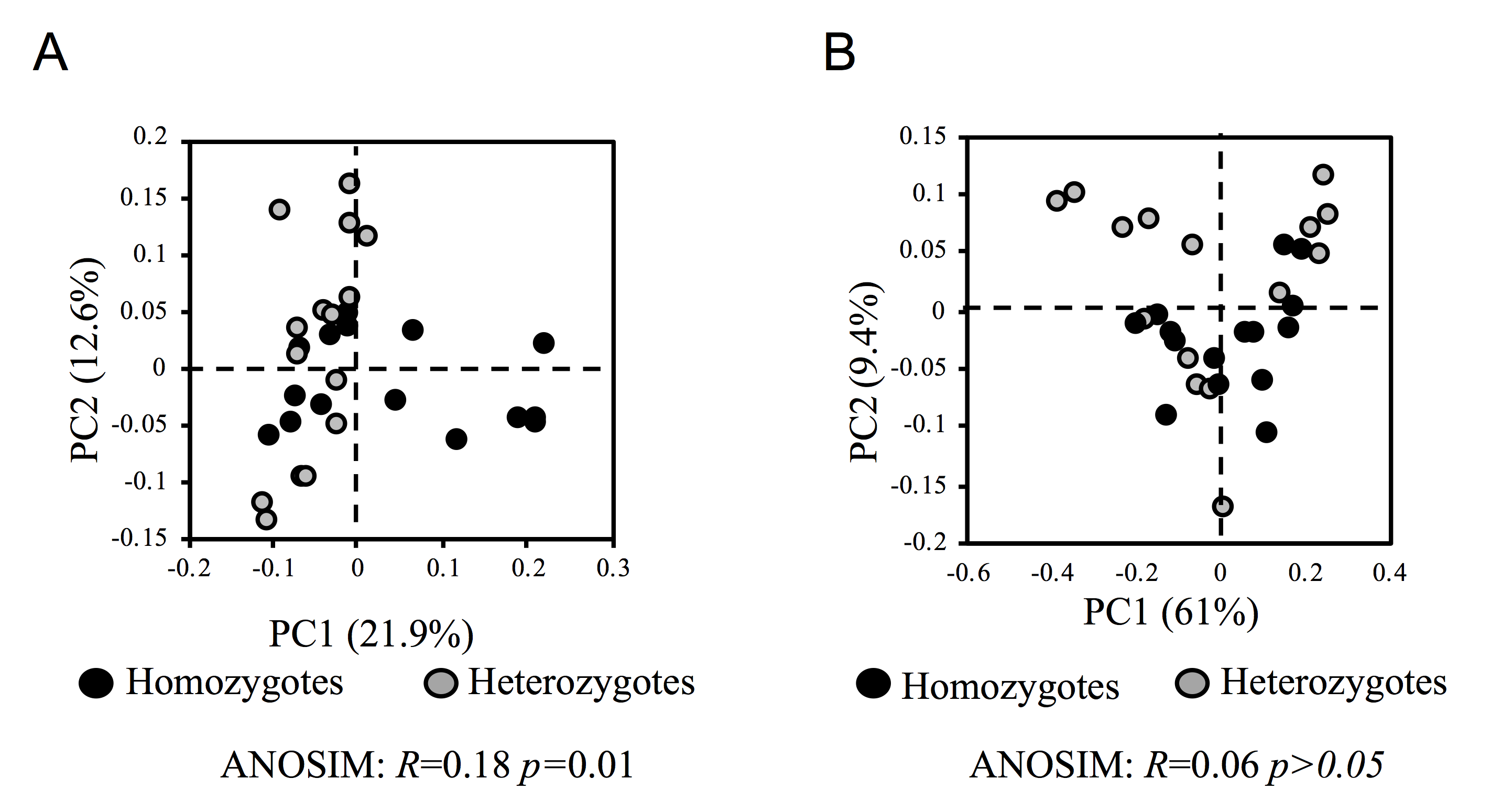

Supplement: S2 Fig — A) PCoA of beta-diversity comparison of overall community between MHC homozygote and MHC heterozygote animals based on Unweighted Unifrac analysis (ANOSIM: R = 0.18; P = 0.01; no. of permutations = 99). B) PCoA of beta-diversity comparison of core community between MHC homozygote and MHC heterozygote animals based on Weighted Unifrac analysis (ANOSIM: R = 0.06; P > 0.05; no. of permutations = 99). (TIFF) [file pone.0215946.s002.tiff]
