# Supplementary material for: Does MHC heterozygosity influence microbiota form and function?
Source: PLoS One. 2019 May 16;14(5):e0215946. doi: 10.1371/journal.pone.0215946 (PMC6522005; doi:10.1371/journal.pone.0215946)
Supplement: S1 Table — The positive log2FoldChange values indicate that the taxa enriched in MHC homozygotes, while negative values indicate the taxa enriched in MHC heterozygotes. (DOCX) [file pone.0215946.s003.docx]

**S1 Table**. Differential enrichment of specific taxa within the overall microbiota of MHC homozygote and MHC heterozygote animals, which are represented as log_2_FoldChange values with *P* < 0.05, Benjamini-Hochberg corrected. The positive log_2_FoldChange values indicate that the taxa enriched in MHC homozygotes, while negative values indicate the taxa enriched in MHC heterozygotes.

| OTU ID | log2FoldChange | *P*-value (Benjamini-Hochberg adjusted) | taxonomy |
| --- | --- | --- | --- |
| 839215 | -4.89 | 9.12E-09 | k__Bacteria; p__Firmicutes; c__Clostridia; o__Clostridiales; f__Lachnospiraceae; NA; NA |
| 279107 | -4.43 | 8.89E-09 | k__Bacteria; p__Firmicutes; c__Clostridia; o__Clostridiales; f__Lachnospiraceae; g__Anaerostipes; s__ |
| 274021 | -3.55 | 3.11E-10 | k__Bacteria; p__Firmicutes; c__Clostridia; o__Clostridiales; f__Lachnospiraceae; g__; s__ |
| 258165 | -3.43 | 1.21E-05 | k__Bacteria; p__Firmicutes; c__Clostridia; o__Clostridiales; f__; g__; s__ |
| 2645483 | -2.92 | 2.65E-05 | k__Bacteria; p__Firmicutes; c__Clostridia; o__Clostridiales; NA; NA; NA |
| 4364242 | -2.76 | 2.41E-05 | k__Bacteria; p__Firmicutes; c__Clostridia; o__Clostridiales; NA; NA; NA |
| 259434 | -2.65 | 3.75E-07 | k__Bacteria; p__Firmicutes; c__Clostridia; o__Clostridiales; f__Lachnospiraceae; NA; NA |
| 193288 | -2.46 | 2.82E-06 | k__Bacteria; p__Firmicutes; c__Clostridia; o__Clostridiales; f__; g__; s__ |
| 198604 | -2.42 | 0.0001 | k__Bacteria; p__Bacteroidetes; c__Bacteroidia; o__Bacteroidales; f__S24-7; g__; s__ |
| 234912 | -2.41 | 0.0003 | k__Bacteria; p__Firmicutes; c__Clostridia; o__Clostridiales; NA; NA; NA |
| 179265 | -2.35 | 6.29E-05 | k__Bacteria; p__Firmicutes; c__Clostridia; o__Clostridiales; f__; g__; s__ |
| 182906 | -2.35 | 0.0003 | k__Bacteria; p__Firmicutes; c__Clostridia; o__Clostridiales; NA; NA; NA |
| 1802717 | -2.31 | 7.93E-05 | k__Bacteria; p__Bacteroidetes; c__Bacteroidia; o__Bacteroidales; f__S24-7; g__; s__ |
| 184966 | -2.27 | 0.0003 | k__Bacteria; p__Firmicutes; c__Clostridia; o__Clostridiales; NA; NA; NA |
| 233991 | -2.24 | 5.55E-05 | k__Bacteria; p__Firmicutes; c__Clostridia; o__Clostridiales; f__Lachnospiraceae; g__; s__ |
| 202419 | -2.17 | 0.0013 | k__Bacteria; p__Firmicutes; c__Clostridia; o__Clostridiales; NA; NA; NA |
| 214919 | -2.12 | 0.0055 | k__Bacteria; p__Firmicutes; c__Bacilli; o__Turicibacterales; f__Turicibacteraceae; g__Turicibacter; s__ |
| 175758 | -1.98 | 0.0228 | k__Bacteria; p__Firmicutes; c__Clostridia; o__Clostridiales; NA; NA; NA |
| 421538 | -1.98 | 0.0046 | k__Bacteria; p__Firmicutes; c__Clostridia; o__Clostridiales; f__Lachnospiraceae; NA; NA |
| 195433 | -1.98 | 0.0046 | k__Bacteria; p__Firmicutes; c__Clostridia; o__Clostridiales; NA; NA; NA |
| 189024 | -1.95 | 0.0003 | k__Bacteria; p__Firmicutes; c__Clostridia; o__Clostridiales; NA; NA; NA |
| 318162 | -1.95 | 0.0003 | k__Bacteria; p__Firmicutes; c__Clostridia; o__Clostridiales; NA; NA; NA |
| 183106 | -1.94 | 0.0009 | k__Bacteria; p__Bacteroidetes; c__Bacteroidia; o__Bacteroidales; f__S24-7; g__; s__ |
| 193509 | -1.93 | 4.93E-06 | k__Bacteria; p__Firmicutes; c__Clostridia; o__Clostridiales; f__Lachnospiraceae; g__Dorea; NA |
| 829401 | -1.91 | 0.0075 | k__Bacteria; p__Firmicutes; c__Clostridia; o__Clostridiales; NA; NA; NA |
| 180879 | -1.91 | 0.0027 | k__Bacteria; p__Firmicutes; c__Clostridia; o__Clostridiales; f__Ruminococcaceae; g__Oscillospira; s__ |
| 309302 | -1.87 | 0.0009 | k__Bacteria; p__Firmicutes; c__Clostridia; o__Clostridiales; f__Ruminococcaceae; g__Oscillospira; s__ |
| 339791 | -1.81 | 0.0015 | k__Bacteria; p__Firmicutes; c__Clostridia; o__Clostridiales; f__Lachnospiraceae; g__Coprococcus; NA |
| 1105958 | -1.79 | 0.0004 | k__Bacteria; p__Firmicutes; c__Clostridia; o__Clostridiales; NA; NA; NA |
| 215317 | -1.76 | 0.0004 | k__Bacteria; p__Firmicutes; c__Clostridia; o__Clostridiales; f__Lachnospiraceae; NA; NA |
| 276312 | -1.73 | 0.0094 | k__Bacteria; p__Firmicutes; c__Clostridia; o__Clostridiales; NA; NA; NA |
| 178779 | -1.73 | 6.67E-05 | k__Bacteria; p__Firmicutes; c__Clostridia; o__Clostridiales; NA; NA; NA |
| 263899 | -1.71 | 0.0032 | k__Bacteria; p__Firmicutes; c__Clostridia; o__Clostridiales; NA; NA; NA |
| 343264 | -1.70 | 0.0078 | k__Bacteria; p__Firmicutes; c__Clostridia; o__Clostridiales; f__; g__; s__ |
| 375106 | -1.65 | 0.0002 | k__Bacteria; p__Firmicutes; c__Clostridia; o__Clostridiales; f__Ruminococcaceae; NA; NA |
| 264101 | -1.62 | 0.0044 | k__Bacteria; p__Firmicutes; c__Clostridia; o__Clostridiales; f__Ruminococcaceae; g__Oscillospira; s__ |
| 276509 | -1.62 | 0.0014 | k__Bacteria; p__Bacteroidetes; c__Bacteroidia; o__Bacteroidales; f__S24-7; g__; s__ |
| 261511 | -1.60 | 0.0003 | k__Bacteria; p__Firmicutes; c__Clostridia; o__Clostridiales; f__; g__; s__ |
| 176691 | -1.60 | 0.0012 | k__Bacteria; p__Firmicutes; c__Clostridia; o__Clostridiales; f__; g__; s__ |
| 275676 | -1.57 | 0.0017 | k__Bacteria; p__Firmicutes; c__Clostridia; o__Clostridiales; NA; NA; NA |
| 182079 | -1.55 | 0.0144 | k__Bacteria; p__Firmicutes; c__Clostridia; o__Clostridiales; NA; NA; NA |
| 1110253 | -1.55 | 0.0037 | k__Bacteria; p__Firmicutes; c__Clostridia; o__Clostridiales; f__Ruminococcaceae; g__Oscillospira; s__ |
| 265483 | -1.54 | 0.0393 | k__Bacteria; p__Firmicutes; c__Clostridia; o__Clostridiales; f__Ruminococcaceae; g__Oscillospira; s__ |
| 170561 | -1.51 | 0.0032 | k__Bacteria; p__Firmicutes; c__Clostridia; o__Clostridiales; f__Lachnospiraceae; NA; NA |
| 267470 | -1.50 | 0.0007 | k__Bacteria; p__Firmicutes; c__Clostridia; o__Clostridiales; NA; NA; NA |
| 213896 | -1.48 | 0.0139 | k__Bacteria; p__Firmicutes; c__Clostridia; o__Clostridiales; NA; NA; NA |
| 174698 | -1.48 | 0.0223 | k__Bacteria; p__Firmicutes; c__Clostridia; o__Clostridiales; NA; NA; NA |
| 274697 | -1.44 | 0.0237 | k__Bacteria; p__Firmicutes; c__Clostridia; o__Clostridiales; NA; NA; NA |
| 178959 | -1.43 | 0.0036 | k__Bacteria; p__Firmicutes; c__Clostridia; o__Clostridiales; f__Ruminococcaceae; g__Oscillospira; s__ |
| 273806 | -1.43 | 0.0170 | k__Bacteria; p__Firmicutes; c__Clostridia; o__Clostridiales; f__; g__; s__ |
| 259511 | -1.40 | 0.0128 | k__Bacteria; p__Firmicutes; c__Clostridia; o__Clostridiales; f__; g__; s__ |
| 188548 | -1.37 | 0.0037 | k__Bacteria; p__Firmicutes; c__Clostridia; o__Clostridiales; NA; NA; NA |
| 4480557 | -1.36 | 0.0125 | k__Bacteria; p__Firmicutes; c__Clostridia; o__Clostridiales; f__Lachnospiraceae; g__; s__ |
| 177533 | -1.35 | 0.0089 | k__Bacteria; p__Bacteroidetes; c__Bacteroidia; o__Bacteroidales; f__S24-7; g__; s__ |
| 170185 | -1.35 | 0.0045 | k__Bacteria; p__Firmicutes; c__Clostridia; o__Clostridiales; f__Ruminococcaceae; NA; NA |
| 269673 | -1.34 | 0.0039 | k__Bacteria; p__Bacteroidetes; c__Bacteroidia; o__Bacteroidales; f__S24-7; g__; s__ |
| 2897332 | -1.34 | 0.0001 | k__Bacteria; p__Firmicutes; c__Clostridia; o__Clostridiales; f__Lachnospiraceae; g__[Ruminococcus]; s__gnavus |
| 271403 | -1.33 | 0.0096 | k__Bacteria; p__Firmicutes; c__Clostridia; o__Clostridiales; f__; g__; s__ |
| 182016 | -1.32 | 0.0032 | k__Bacteria; p__Firmicutes; c__Clostridia; o__Clostridiales; f__Lachnospiraceae; g__Coprococcus; s__ |
| 331786 | -1.26 | 0.0153 | k__Bacteria; p__Firmicutes; c__Clostridia; o__Clostridiales; NA; NA; NA |
| 265940 | -1.23 | 0.0431 | k__Bacteria; p__Firmicutes; c__Clostridia; o__Clostridiales; f__Ruminococcaceae; g__Ruminococcus; s__ |
| 271830 | -1.20 | 0.0484 | k__Bacteria; p__Bacteroidetes; c__Bacteroidia; o__Bacteroidales; f__S24-7; g__; s__ |
| 272341 | -1.19 | 0.0206 | k__Bacteria; p__Firmicutes; c__Clostridia; o__Clostridiales; NA; NA; NA |
| 271219 | -1.18 | 0.0092 | k__Bacteria; p__Firmicutes; c__Clostridia; o__Clostridiales; f__; g__; s__ |
| 258717 | -1.17 | 0.0176 | k__Bacteria; p__Firmicutes; c__Clostridia; o__Clostridiales; f__Lachnospiraceae; g__Dorea; s__ |
| 196571 | -1.17 | 0.0147 | k__Bacteria; p__Firmicutes; c__Clostridia; o__Clostridiales; f__; g__; s__ |
| 174819 | -1.16 | 0.0062 | k__Bacteria; p__Firmicutes; c__Clostridia; o__Clostridiales; NA; NA; NA |
| 172464 | -1.16 | 0.0158 | k__Bacteria; p__Firmicutes; c__Clostridia; o__Clostridiales; NA; NA; NA |
| 178480 | -1.14 | 0.0182 | k__Bacteria; p__Firmicutes; c__Clostridia; o__Clostridiales; f__Ruminococcaceae; g__Ruminococcus; s__ |
| 262102 | -1.11 | 0.0203 | k__Bacteria; p__Bacteroidetes; c__Bacteroidia; o__Bacteroidales; f__S24-7; g__; s__ |
| 185923 | -1.10 | 0.0358 | k__Bacteria; p__Firmicutes; c__Clostridia; o__Clostridiales; NA; NA; NA |
| 175573 | -1.10 | 0.0192 | k__Bacteria; p__Firmicutes; c__Clostridia; o__Clostridiales; f__; g__; s__ |
| 276016 | -1.09 | 0.0135 | k__Bacteria; p__Firmicutes; c__Clostridia; o__Clostridiales; f__Lachnospiraceae; g__[Ruminococcus]; s__gnavus |
| 356055 | -1.06 | 0.0213 | k__Bacteria; p__Firmicutes; c__Clostridia; o__Clostridiales; f__Ruminococcaceae; g__Oscillospira; s__ |
| 195445 | -1.06 | 0.0078 | k__Bacteria; p__Firmicutes; c__Clostridia; o__Clostridiales; NA; NA; NA |
| 265793 | -1.06 | 0.0258 | k__Bacteria; p__Firmicutes; c__Clostridia; o__Clostridiales; f__Ruminococcaceae; g__Oscillospira; s__ |
| 188299 | -1.02 | 0.0308 | k__Bacteria; p__Firmicutes; c__Clostridia; o__Clostridiales; f__Lachnospiraceae; g__; s__ |
| 272576 | -1.00 | 0.0119 | k__Bacteria; p__Firmicutes; c__Clostridia; o__Clostridiales; f__Ruminococcaceae; g__Oscillospira; s__ |
| 345330 | -0.99 | 0.0405 | k__Bacteria; p__Bacteroidetes; c__Bacteroidia; o__Bacteroidales; f__S24-7; g__; s__ |
| 275974 | -0.97 | 0.0303 | k__Bacteria; p__Actinobacteria; c__Coriobacteriia; o__Coriobacteriales; f__Coriobacteriaceae; g__Adlercreutzia; s__ |
| 2797565 | -0.97 | 0.0076 | k__Bacteria; p__Firmicutes; c__Clostridia; o__Clostridiales; f__[Mogibacteriaceae]; g__; s__ |
| 182033 | -0.96 | 0.0112 | k__Bacteria; p__Firmicutes; c__Clostridia; o__Clostridiales; f__Ruminococcaceae; g__Oscillospira; s__ |
| 178735 | -0.96 | 0.0071 | k__Bacteria; p__Actinobacteria; c__Coriobacteriia; o__Coriobacteriales; f__Coriobacteriaceae; g__Adlercreutzia; s__ |
| 232283 | -0.96 | 0.0436 | k__Bacteria; p__Firmicutes; c__Clostridia; o__Clostridiales; f__Lachnospiraceae; g__Coprococcus; s__ |
| 169234 | -0.96 | 0.0320 | k__Bacteria; p__Firmicutes; c__Clostridia; o__Clostridiales; f__Lachnospiraceae; NA; NA |
| 261142 | -0.95 | 0.0281 | k__Bacteria; p__Firmicutes; c__Clostridia; o__Clostridiales; f__; g__; s__ |
| 389529 | -0.95 | 0.0211 | k__Bacteria; p__Firmicutes; c__Clostridia; o__Clostridiales; f__Ruminococcaceae; NA; NA |
| 276837 | -0.93 | 0.0406 | k__Bacteria; p__Firmicutes; c__Clostridia; o__Clostridiales; NA; NA; NA |
| 167034 | -0.92 | 0.0060 | k__Bacteria; p__Firmicutes; c__Clostridia; o__Clostridiales; f__Ruminococcaceae; g__Oscillospira; s__ |
| 165046 | -0.91 | 0.0353 | k__Bacteria; p__Firmicutes; c__Clostridia; o__Clostridiales; f__Ruminococcaceae; g__Oscillospira; s__ |
| 265106 | -0.81 | 0.0197 | k__Bacteria; p__Actinobacteria; c__Coriobacteriia; o__Coriobacteriales; f__Coriobacteriaceae; g__Adlercreutzia; s__ |
| 4481359 | 0.73 | 0.0422 | k__Bacteria; p__Firmicutes; c__Clostridia; o__Clostridiales; f__Lachnospiraceae; g__; s__ |
| 268720 | 0.77 | 0.0285 | k__Bacteria; p__Firmicutes; c__Clostridia; o__Clostridiales; f__Ruminococcaceae; g__Ruminococcus; s__ |
| 188138 | 0.77 | 0.0338 | k__Bacteria; p__Firmicutes; c__Clostridia; o__Clostridiales; f__Lachnospiraceae; NA; NA |
| 176118 | 0.81 | 0.0497 | k__Bacteria; p__Firmicutes; c__Clostridia; o__Clostridiales; f__Ruminococcaceae; g__Oscillospira; s__ |
| 186052 | 0.81 | 0.0255 | k__Bacteria; p__Firmicutes; c__Clostridia; o__Clostridiales; f__Lachnospiraceae; g__; s__ |
| 179059 | 0.85 | 0.0168 | k__Bacteria; p__Firmicutes; c__Clostridia; o__Clostridiales; NA; NA; NA |
| 259382 | 0.87 | 0.0288 | k__Bacteria; p__Firmicutes; c__Clostridia; o__Clostridiales; f__Lachnospiraceae; NA; NA |
| 341223 | 0.88 | 0.0390 | k__Bacteria; p__Firmicutes; c__Clostridia; o__Clostridiales; f__Lachnospiraceae; g__; s__ |
| 275293 | 0.88 | 0.0371 | k__Bacteria; p__Firmicutes; c__Clostridia; o__Clostridiales; NA; NA; NA |
| 262326 | 0.91 | 0.0352 | k__Bacteria; p__Firmicutes; c__Clostridia; o__Clostridiales; f__Lachnospiraceae; NA; NA |
| 337407 | 0.94 | 0.0443 | k__Bacteria; p__Firmicutes; c__Clostridia; o__Clostridiales; NA; NA; NA |
| 214835 | 0.95 | 0.0213 | k__Bacteria; p__Firmicutes; c__Clostridia; o__Clostridiales; NA; NA; NA |
| 228730 | 0.95 | 0.0370 | k__Bacteria; p__Bacteroidetes; c__Bacteroidia; o__Bacteroidales; f__S24-7; g__; s__ |
| 293754 | 1.00 | 0.0480 | k__Bacteria; p__Firmicutes; c__Clostridia; o__Clostridiales; NA; NA; NA |
| 1902400 | 1.04 | 0.0304 | k__Bacteria; p__Firmicutes; c__Clostridia; o__Clostridiales; f__Lachnospiraceae; NA; NA |
| 176236 | 1.04 | 0.0078 | k__Bacteria; p__Firmicutes; c__Clostridia; o__Clostridiales; NA; NA; NA |
| 189666 | 1.05 | 0.0354 | k__Bacteria; p__Firmicutes; c__Clostridia; o__Clostridiales; NA; NA; NA |
| 313095 | 1.05 | 0.0162 | k__Bacteria; p__Firmicutes; c__Clostridia; o__Clostridiales; NA; NA; NA |
| 174667 | 1.06 | 0.0050 | k__Bacteria; p__Firmicutes; c__Clostridia; o__Clostridiales; f__Lachnospiraceae; g__; s__ |
| 259912 | 1.07 | 0.0065 | k__Bacteria; p__Firmicutes; c__Clostridia; o__Clostridiales; NA; NA; NA |
| 262104 | 1.07 | 0.0385 | k__Bacteria; p__Firmicutes; c__Clostridia; o__Clostridiales; NA; NA; NA |
| 180466 | 1.07 | 0.0116 | k__Bacteria; p__Firmicutes; c__Clostridia; o__Clostridiales; f__Lachnospiraceae; NA; NA |
| 263138 | 1.10 | 0.0061 | k__Bacteria; p__Firmicutes; c__Clostridia; o__Clostridiales; f__Lachnospiraceae; NA; NA |
| 170335 | 1.10 | 0.0442 | k__Bacteria; p__Bacteroidetes; c__Bacteroidia; o__Bacteroidales; f__[Odoribacteraceae]; g__Odoribacter; s__ |
| 1136443 | 1.10 | 0.0364 | k__Bacteria; p__Deferribacteres; c__Deferribacteres; o__Deferribacterales; f__Deferribacteraceae; g__Mucispirillum; s__schaedleri |
| 38415 | 1.12 | 0.0299 | k__Bacteria; p__Firmicutes; c__Clostridia; o__Clostridiales; f__Lachnospiraceae; NA; NA |
| 275138 | 1.13 | 0.0177 | k__Bacteria; p__Firmicutes; c__Clostridia; o__Clostridiales; NA; NA; NA |
| 277332 | 1.14 | 0.0108 | k__Bacteria; p__Firmicutes; c__Clostridia; o__Clostridiales; f__; g__; s__ |
| 318764 | 1.15 | 0.0350 | k__Bacteria; p__Firmicutes; c__Bacilli; o__Lactobacillales; f__Lactobacillaceae; g__Lactobacillus; NA |
| 180584 | 1.18 | 0.0289 | k__Bacteria; p__Firmicutes; c__Clostridia; o__Clostridiales; NA; NA; NA |
| 295075 | 1.18 | 0.0445 | k__Bacteria; p__Firmicutes; c__Clostridia; o__Clostridiales; f__Lachnospiraceae; g__[Ruminococcus]; s__gnavus |
| 346648 | 1.19 | 0.0214 | k__Bacteria; p__Firmicutes; c__Clostridia; o__Clostridiales; f__Lachnospiraceae; g__; s__ |
| 178114 | 1.19 | 0.0085 | k__Bacteria; p__Bacteroidetes; c__Bacteroidia; o__Bacteroidales; f__S24-7; g__; s__ |
| 272092 | 1.19 | 0.0390 | k__Bacteria; p__Firmicutes; c__Clostridia; o__Clostridiales; f__; g__; s__ |
| 267752 | 1.20 | 0.0256 | k__Bacteria; p__Firmicutes; c__Clostridia; o__Clostridiales; NA; NA; NA |
| 191811 | 1.24 | 0.0150 | k__Bacteria; p__Firmicutes; c__Clostridia; o__Clostridiales; f__; g__; s__ |
| 260553 | 1.25 | 0.0035 | k__Bacteria; p__Firmicutes; c__Clostridia; o__Clostridiales; f__Lachnospiraceae; NA; NA |
| 260666 | 1.26 | 0.0381 | k__Bacteria; p__Firmicutes; c__Clostridia; o__Clostridiales; f__Lachnospiraceae; NA; NA |
| 264660 | 1.26 | 0.0422 | k__Bacteria; p__Firmicutes; c__Clostridia; o__Clostridiales; f__; g__; s__ |
| 335267 | 1.26 | 0.0027 | k__Bacteria; p__Firmicutes; c__Clostridia; o__Clostridiales; NA; NA; NA |
| 194822 | 1.29 | 0.0002 | k__Bacteria; p__Firmicutes; c__Clostridia; o__Clostridiales; f__Lachnospiraceae; NA; NA |
| 319134 | 1.32 | 0.0087 | k__Bacteria; p__Firmicutes; c__Clostridia; o__Clostridiales; f__; g__; s__ |
| 309480 | 1.32 | 0.0105 | k__Bacteria; p__Firmicutes; c__Clostridia; o__Clostridiales; f__Lachnospiraceae; g__Dorea; NA |
| 172339 | 1.32 | 0.0019 | k__Bacteria; p__Firmicutes; c__Clostridia; o__Clostridiales; f__Ruminococcaceae; g__Butyricicoccus; s__pullicaecorum |
| 183907 | 1.32 | 0.0156 | k__Bacteria; p__Firmicutes; c__Clostridia; o__Clostridiales; NA; NA; NA |
| 174847 | 1.32 | 0.0107 | k__Bacteria; p__Firmicutes; c__Clostridia; o__Clostridiales; f__; g__; s__ |
| 188116 | 1.39 | 0.0003 | k__Bacteria; p__Firmicutes; c__Clostridia; o__Clostridiales; f__Lachnospiraceae; NA; NA |
| 190460 | 1.39 | 0.0045 | k__Bacteria; p__Firmicutes; c__Clostridia; o__Clostridiales; NA; NA; NA |
| 3919792 | 1.40 | 0.0357 | k__Bacteria; p__Firmicutes; c__Clostridia; o__Clostridiales; f__; g__; s__ |
| 262537 | 1.40 | 0.0011 | k__Bacteria; p__Firmicutes; c__Clostridia; o__Clostridiales; f__Lachnospiraceae; g__[Ruminococcus]; s__gnavus |
| 199391 | 1.41 | 0.0142 | k__Bacteria; p__Firmicutes; c__Clostridia; o__Clostridiales; NA; NA; NA |
| 317389 | 1.45 | 0.0056 | k__Bacteria; p__Firmicutes; c__Clostridia; o__Clostridiales; NA; NA; NA |
| 191067 | 1.48 | 0.0033 | k__Bacteria; p__Firmicutes; c__Clostridia; o__Clostridiales; NA; NA; NA |
| 259275 | 1.49 | 0.0331 | k__Bacteria; p__Firmicutes; c__Clostridia; o__Clostridiales; f__Ruminococcaceae; g__Oscillospira; s__ |
| 192365 | 1.50 | 0.0034 | k__Bacteria; p__Firmicutes; c__Clostridia; o__Clostridiales; f__; g__; s__ |
| 289177 | 1.51 | 0.0472 | k__Bacteria; p__Firmicutes; c__Clostridia; o__Clostridiales; f__; g__; s__ |
| 189585 | 1.54 | 0.0161 | k__Bacteria; p__Firmicutes; c__Clostridia; o__Clostridiales; f__; g__; s__ |
| 199731 | 1.54 | 0.0275 | k__Bacteria; p__Firmicutes; c__Clostridia; o__Clostridiales; NA; NA; NA |
| 181719 | 1.56 | 0.0193 | k__Bacteria; p__Bacteroidetes; c__Bacteroidia; o__Bacteroidales; f__Bacteroidaceae; g__Bacteroides; s__ |
| 335632 | 1.58 | 0.0036 | k__Bacteria; p__Firmicutes; c__Clostridia; o__Clostridiales; NA; NA; NA |
| 322564 | 1.59 | 0.0126 | k__Bacteria; p__Firmicutes; c__Clostridia; o__Clostridiales; NA; NA; NA |
| 186645 | 1.62 | 0.0003 | k__Bacteria; p__Firmicutes; c__Clostridia; o__Clostridiales; NA; NA; NA |
| 347908 | 1.65 | 0.0030 | k__Bacteria; p__Firmicutes; c__Clostridia; o__Clostridiales; f__Lachnospiraceae; NA; NA |
| 178068 | 1.65 | 0.0289 | k__Bacteria; p__Bacteroidetes; c__Bacteroidia; o__Bacteroidales; f__S24-7; g__; s__ |
| 323595 | 1.67 | 0.0044 | k__Bacteria; p__Firmicutes; c__Clostridia; o__Clostridiales; NA; NA; NA |
| 195711 | 1.67 | 0.0163 | k__Bacteria; p__Firmicutes; c__Clostridia; o__Clostridiales; NA; NA; NA |
| 327739 | 1.70 | 0.0022 | k__Bacteria; p__Firmicutes; c__Clostridia; o__Clostridiales; f__Lachnospiraceae; g__; s__ |
| 177927 | 1.73 | 0.0019 | k__Bacteria; p__Firmicutes; c__Clostridia; o__Clostridiales; f__; g__; s__ |
| 797021 | 1.73 | 0.0012 | k__Bacteria; p__Firmicutes; c__Clostridia; o__Clostridiales; NA; NA; NA |
| 327656 | 1.73 | 0.0008 | k__Bacteria; p__Firmicutes; c__Clostridia; o__Clostridiales; NA; NA; NA |
| 178787 | 1.76 | 0.0038 | k__Bacteria; p__Firmicutes; c__Clostridia; o__Clostridiales; NA; NA; NA |
| 267914 | 1.76 | 0.0035 | k__Bacteria; p__Firmicutes; c__Clostridia; o__Clostridiales; f__; g__; s__ |
| 274148 | 1.78 | 0.0035 | k__Bacteria; p__Firmicutes; c__Clostridia; o__Clostridiales; NA; NA; NA |
| 310407 | 1.80 | 0.0004 | k__Bacteria; p__Firmicutes; c__Clostridia; o__Clostridiales; f__Lachnospiraceae; NA; NA |
| 266860 | 1.81 | 0.0169 | k__Bacteria; p__Bacteroidetes; c__Bacteroidia; o__Bacteroidales; f__S24-7; g__; s__ |
| 2897325 | 1.82 | 1.47E-06 | k__Bacteria; p__Proteobacteria; c__Deltaproteobacteria; o__Desulfovibrionales; f__Desulfovibrionaceae; g__Bilophila; s__ |
| 197568 | 1.92 | 0.0031 | k__Bacteria; p__Firmicutes; c__Clostridia; o__Clostridiales; f__; g__; s__ |
| 340853 | 2.01 | 0.0002 | k__Bacteria; p__Firmicutes; c__Clostridia; o__Clostridiales; f__Lachnospiraceae; g__; s__ |
| 177022 | 2.05 | 0.0016 | k__Bacteria; p__Firmicutes; c__Clostridia; o__Clostridiales; f__; g__; s__ |
| 309361 | 2.17 | 0.0003 | k__Bacteria; p__Firmicutes; c__Clostridia; o__Clostridiales; f__Ruminococcaceae; g__Oscillospira; s__ |
| 264345 | 2.23 | 0.0085 | k__Bacteria; p__Firmicutes; c__Clostridia; o__Clostridiales; f__Lachnospiraceae; NA; NA |
| 4397402 | 2.28 | 0.0077 | k__Bacteria; p__Firmicutes; c__Bacilli; o__Lactobacillales; f__Lactobacillaceae; g__Lactobacillus; s__ |
| 196315 | 2.38 | 7.79E-05 | k__Bacteria; p__Firmicutes; c__Clostridia; o__Clostridiales; f__Clostridiaceae; g__SMB53; s__ |
| 227967 | 2.38 | 8.57E-05 | k__Bacteria; p__Firmicutes; c__Clostridia; o__Clostridiales; NA; NA; NA |
| 228140 | 2.56 | 1.74E-05 | k__Bacteria; p__Firmicutes; c__Clostridia; o__Clostridiales; f__[Mogibacteriaceae]; g__; s__ |
| 273208 | 2.57 | 0.0008 | k__Bacteria; p__Bacteroidetes; c__Bacteroidia; o__Bacteroidales; f__S24-7; g__; s__ |
| 320912 | 2.57 | 0.0001 | k__Bacteria; p__Firmicutes; c__Clostridia; o__Clostridiales; f__Lachnospiraceae; NA; NA |
| 188078 | 2.57 | 0.0002 | k__Bacteria; p__Firmicutes; c__Clostridia; o__Clostridiales; f__Lachnospiraceae; g__; s__ |
| 323154 | 2.59 | 2.01E-05 | k__Bacteria; p__Firmicutes; c__Clostridia; o__Clostridiales; f__Lachnospiraceae; g__; s__ |
| 266343 | 2.59 | 1.40E-07 | k__Bacteria; p__Firmicutes; c__Clostridia; o__Clostridiales; f__Lachnospiraceae; NA; NA |
| 318819 | 2.61 | 2.55E-05 | k__Bacteria; p__Firmicutes; c__Clostridia; o__Clostridiales; NA; NA; NA |
| 261590 | 3.57 | 4.69E-06 | k__Bacteria; p__Firmicutes; c__Clostridia; o__Clostridiales; NA; NA; NA |
| 189407 | 3.77 | 2.45E-07 | k__Bacteria; p__Firmicutes; c__Clostridia; o__Clostridiales; f__Clostridiaceae; g__SMB53; s__ |
| 346291 | 4.06 | 1.01E-07 | k__Bacteria; p__Firmicutes; c__Clostridia; o__Clostridiales; NA; NA; NA |
| 261178 | 4.12 | 2.06E-09 | k__Bacteria; p__Firmicutes; c__Clostridia; o__Clostridiales; f__Lachnospiraceae; g__; s__ |
| 318732 | 4.14 | 2.77E-07 | k__Bacteria; p__Firmicutes; c__Clostridia; o__Clostridiales; NA; NA; NA |
| 681370 | 4.54 | 6.65E-10 | k__Bacteria; p__Actinobacteria; c__Actinobacteria; o__Bifidobacteriales; f__Bifidobacteriaceae; g__Bifidobacterium; s__pseudolongum |
| 167204 | 6.51 | 6.02E-13 | k__Bacteria; p__Firmicutes; c__Clostridia; o__Clostridiales; NA; NA; NA |
