# Supplementary material for: Does MHC heterozygosity influence microbiota form and function?
Source: PLoS One. 2019 May 16;14(5):e0215946. doi: 10.1371/journal.pone.0215946 (PMC6522005; doi:10.1371/journal.pone.0215946)
Supplement: S3 Table — The score represents the extent to which OTUs in a given sample are related to their sequenced genomes. (DOCX) [file pone.0215946.s005.docx]

**S3 Table.** Summary of the weighted Nearest Sequenced Taxon Index (weighted NSTI) scores of each of the samples used in this study. The score represents the extent to which OTUs in a given sample are related to their sequenced genomes.

| **Sample** | **Genotype** | **Weighted NSTI score** |
| --- | --- | --- |
| BB70 | H2*^bb^* | 0.24 |
| BB71 | H2*^bb^* | 0.25 |
| BB72 | H2*^bb^* | 0.20 |
| BB73 | H2*^bb^* | 0.24 |
| BB74 | H2*^bb^* | 0.18 |
| BD158 | H2*^bd^* | 0.18 |
| BD159 | H2*^bd^* | 0.23 |
| BD160 | H2*^bd^* | 0.24 |
| BD161 | H2*^bd^* | 0.25 |
| BD162 | H2*^bd^* | 0.22 |
| DD89 | H2*^dd^* | 0.18 |
| DD90 | H2*^dd^* | 0.20 |
| DD91 | H2*^dd^* | 0.20 |
| DD92 | H2*^dd^* | 0.25 |
| DD93 | H2*^dd^* | 0.20 |
| DK141 | H2*^dk^* | 0.23 |
| DK142 | H2*^dk^* | 0.27 |
| DK143 | H2*^dk^* | 0.29 |
| DK145 | H2*^dk^* | 0.18 |
| DK146 | H2*^dk^* | 0.19 |
| KB131 | H2*^kb^* | 0.17 |
| KB132 | H2*^kb^* | 0.21 |
| KB133 | H2*^kb^* | 0.18 |
| KB134 | H2*^kb^* | 0.19 |
| KB135 | H2*^kb^* | 0.30 |
| KK74 | H2*^kk^* | 0.18 |
| KK75 | H2*^kk^* | 0.25 |
| KK76 | H2*^kk^* | 0.19 |
| KK77 | H2*^kk^* | 0.19 |
| KK78 | H2*^kk^* | 0.18 |
